# Interventions for trachoma trichiasis

**DOI:** 10.1002/14651858.CD004008.pub3

**Published:** 2015-11-13

**Authors:** Matthew Burton, Esmael Habtamu, Derek Ho, Emily W Gower

**Affiliations:** London School of Hygiene & Tropical MedicineInternational Centre for Eye HealthKeppel StreetLondonUKWC1E 7HT; London School of Hygiene and Tropical MedicineKeppel StreetLondonUKWC1E 7HT; Imperial CollegeLondonUK; Wake Forest Public Health Sciences and OphthalmologyMedical Center BlvdWinston‐SalemNCUSA27157

**Keywords:** Humans, Anti-Bacterial Agents, Anti-Bacterial Agents/therapeutic use, Chlamydia trachomatis, Entropion, Entropion/etiology, Entropion/surgery, Eyelid Diseases, Eyelid Diseases/surgery, Eyelid Diseases/therapy, Hair Removal, Hair Removal/methods, Randomized Controlled Trials as Topic, Trachoma, Trachoma/surgery, Trachoma/therapy

## Abstract

**Background:**

Trachoma is the leading infectious cause of blindness. The World Health Organization (WHO) recommends eliminating trachomatous blindness through the SAFE strategy: Surgery for trichiasis, Antibiotic treatment, Facial cleanliness and Environmental hygiene. This is an update of a Cochrane review first published in 2003, and previously updated in 2006.

**Objectives:**

To assess the effects of interventions for trachomatous trichiasis for people living in endemic settings.

**Search methods:**

We searched CENTRAL (which contains the Cochrane Eyes and Vision Group Trials Register) (2015, Issue 4), Ovid MEDLINE, Ovid MEDLINE In‐Process and Other Non‐Indexed Citations, Ovid MEDLINE Daily, Ovid OLDMEDLINE (January 1946 to May 2015), EMBASE (January 1980 to May 2015), the ISRCTN registry (www.isrctn.com/editAdvancedSearch), ClinicalTrials.gov (www.clinicaltrials.gov) and the WHO International Clinical Trials Registry Platform (ICTRP) (www.who.int/ictrp/search/en). We did not use any date or language restrictions in the electronic searches for trials. We last searched the electronic databases on 7 May 2015. We searched the reference lists of included studies to identify further potentially relevant studies. We also contacted authors for details of other relevant studies.

**Selection criteria:**

We included randomised trials of any intervention intended to treat trachomatous trichiasis.

**Data collection and analysis:**

Three review authors independently selected and assessed the trials, including the risk of bias. We contacted trial authors for missing data when necessary. Our primary outcome was post‐operative trichiasis which was defined as any lash touching the globe at three months, one year or two years after surgery.

**Main results:**

Thirteen studies met the inclusion criteria with 8586 participants. Most of the studies were conducted in sub‐Saharan Africa. The majority of the studies were of a low or unclear risk of bias.

Five studies compared different surgical interventions. Most surgical interventions were performed by non‐physician technicians. These trials suggest the most effective surgery is full‐thickness incision of the tarsal plate and rotation of the terminal tarsal strip. Pooled data from two studies suggested that the bilamellar rotation was more effective than unilamellar rotation (OR 0.29, 95% CI 0.16 to 0.50). Use of a lid clamp reduced lid contour abnormalities (OR 0.65, 95% CI 0.44 to 0.98) and granuloma formation (OR 0.67, 95% CI 0.46 to 0.97). Absorbable sutures gave comparable outcomes to silk sutures (OR 0.90, 95% CI 0.68 to 1.20) and were associated with less frequent granuloma formation (OR 0.63, 95% CI 0.40 to 0.99). Epilation was less effective at preventing eyelashes from touching the globe than surgery for mild trichiasis, but had comparable results for vision and corneal change. Peri‐operative azithromycin reduced post‐operative trichiasis; however, the estimate of effect was imprecise and compatible with no effect or increased trichiasis (OR 0.85, 95% CI 0.63 to 1.14; 1954 eyes; 3 studies). Community‐based surgery when compared to health centres increased uptake with comparable outcomes. Surgery performed by ophthalmologists and integrated eye care workers was comparable. Adverse events were typically infrequent or mild and included rare postoperative infections, eyelid contour abnormalities and conjunctival granulomas.

**Authors' conclusions:**

No trials were designed to evaluate whether the interventions for trichiasis prevent blindness as an outcome; however, several found modest improvement in vision following intervention. Certain interventions have been shown to be more effective at eliminating trichiasis. Full‐thickness incision of the tarsal plate and rotation of the lash‐bearing lid margin was found to be the best technique and is preferably delivered in the community. Surgery may be carried out by an ophthalmologist or a trained ophthalmic assistant. Surgery performed with silk or absorbable sutures gave comparable results. Post‐operative azithromycin was found to improve outcomes where overall recurrence was low.

## Plain language summary

**Interventions for trachomatous trichiasis**

**Review question** This review addressed the question: "Which interventions improve the results of the treatment for trichiasis (in‐turned eyelashes) caused by trachoma?"

**Background** Trachoma is the commonest infectious cause of blindness in the world. It is caused by a bacterium called *Chlamydia trachomatis.* This infection causes inflammation and scarring of the surface of the eye, which results in the eyelid turning in (entropion) so that the eyelashes touch the eyeball. This is known as trachomatous trichiasis. The lashes can scratch the corneal surface, leading directly or indirectly (from secondary infections) to corneal opacity. Surgery to correct the eyelid deformity is the main treatment for the late stages of the disease. Most cases of trachomatous trichiasis occur in sub‐Saharan Africa. They are generally treated by nurses with limited surgical training. Unfortunately the results of the surgery can be quite variable, with frequent post‐operative trichiasis reported. Therefore, we wanted to find out what types of surgery and other interventions give the best results in treating this condition.

**Study characteristics** We identified 13 randomised controlled trials. They were all conducted in trachoma‐endemic countries (mostly in sub‐Saharan Africa) with surgical interventions carried out by non‐physician surgeons. Five studies compared different surgical treatments. Three studies investigated whether azithromycin antibiotic treatment after surgery improves the results. One study compared different types of sutures. One study compared surgery to the pulling out of eyelashes (epilation). One study compared the outcomes of treatments provided in the community with hospital care. One study compared the results of surgery performed by eye doctors with those of non‐specialist technicians. The evidence is current to May 2015. Most studies were funded by government research councils or charitable foundations.

**Key results** These trials suggested that the most effective surgery requires full‐thickness incision of the tarsal plate and rotation of the edge of the eyelid. The use of a surgical lid clamp improves eyelid contour outcomes and reduces granuloma formation. Silk and absorbable sutures give comparable results. The addition of azithromycin treatment at the time of surgery may reduce post‐operative trichiasis under certain conditions. Epilation is less effective than surgery at treating trichiasis, but has comparable results for vision and corneal change two years after intervention. Community‐based surgery was more convenient for patients by reducing the time and expense of travelling to a conventional hospital, and it did not increase the risk of complications or recurrence. Surgery performed by ophthalmologists and by integrated eye care workers were both similarly effective. Destroying the lash roots by freezing or electrical ablation appeared to have low success rates and the equipment required is costly and can be difficult to maintain.

**Quality of the evidence** The quality of the evidence from these randomised controlled trials was variable. Most were of a high quality. However, several were relatively small in size and several had potential bias problems due to the method of randomisation and masking.

## Background

### Description of the condition

#### Clinical Features

Trachoma is a form of chronic conjunctivitis caused by *Chlamydia trachomatis*. Following repeated infections, the upper tarsal conjunctiva becomes scarred. As the scar tissue contracts, it shortens the posterior lamella (inner surface) of the upper lid causing the eyelashes to turn in (entropion) and rub against the transparent cornea. This contact between one or more lashes and the surface of the eye is called trichiasis. Trichiasis due to trachoma has a wide spectrum of severity from a single lash touching the eye through to the entire upper lid being rolled in (Rajak 2011). It may result from misdirected or metaplastic eyelashes, in the absence of frank entropion (Rajak 2011). Corneal opacification and the resulting blindness probably develop primarily as a result of this trauma and secondary bacterial corneal infection.

#### Epidemiology

The most recent World Health Organization (WHO) estimates suggest that about 40 million people have active trachoma and about 8.2 million have trichiasis (Mariotti 2009). The signs of active trachoma are usually most frequently found in young children, with males and females equally affected. The scarring complications become evident in later life. Women are usually more frequently affected by trichiasis than men (West 1991). The rate at which the scarring complications of the disease progress varies, possibly reflecting variation in the pressure of *C. trachomatis* infection and immunogenetic predisposition of different populations (Hu 2013). Trachoma is most prevalent in hot, dry areas and is associated with poverty (Emerson 2000). The greatest burden of disease is in sub‐Saharan Africa.

### Description of the intervention

#### Trachoma control

The eradication of blinding trachoma is one of the objectives of the global Vision 2020 programme to eliminate avoidable blindness, led by the WHO and the International Agency for the Prevention of Blindness. In 1997 the WHO launched an initiative on trachoma control based on the 'SAFE' strategy. SAFE stands for Surgery for trichiasis, Antibiotics, Facial cleanliness, and Environmental improvement. Improved facial cleanliness and environmental hygiene are effective at reducing transmission by removing the conditions that promote spread of the disease. Antibiotics reduce the risk of disease transmission by treating the infectious agent. Surgery to correct the lid deformity is the only treatment that is likely to be beneficial in the late stages of the disease; however, once corneal opacification has occurred, management options are very limited. Cochrane reviews of the optimum antibiotic regimen for trachoma (Evans 2011), environmental sanitation (Rabiu 2012), and face‐washing promotion (Ejere 2015), are published on the Cochrane Library.

#### Trichiasis treatment options

The primary aim of treatment for trichiasis is to prevent blindness due to trauma from the lashes abrading the cornea (Rajak 2012). Treatments may be divided into non‐surgical treatments and surgical treatments:

#### Non‐surgical treatments

- epilation (manual removal of eyelashes, usually with forceps);
- eyelid‐taping (to force eyelashes back to correct position)

#### Surgical treatments

Surgical procedures for lash ablation or removal:

- electrolysis (fine needle used to pass electric current to base of lash follicle);
- cryotherapy (freezing treatment to the lash follicles)
- excision of lash‐bearing tissue

A wide variety of surgical options are available for the treatment of upper lid entropion (Rajak 2012), and it is likely that certain operations are more successful than others. In trachoma‐endemic countries the most commonly used procedures are:

- bilamellar tarsal rotation (BLTR): full‐thickness incision through the eyelid, including the scarred tarsal plate, orbicularis oculi and the skin, fixation with everting sutures;

- posterior lamellar tarsal rotation (PLTR)/Trabut: incision through the scarred tarsal plate and conjunctiva only, leaving the skin and orbicularis oculi intact, fixation with everting sutures;

- tarsal advance and rotation: incision of the tarsal plate and rotation of the terminal portion. The upper part of the tarsus is separated from the anterior lamellar, advanced and fixed with sutures.

These techniques are illustrated in the WHO's publication ‐ Trichiasis surgery for trachoma (WHO 2013).

### How the intervention might work

The lash treatments directly remove or ablate the follicles. They do not correct any underlying anatomical abnormality such as entropion and are, therefore, generally not suitable if there is a significant degree of entropion. The lid rotation procedures correct the underlying entropion by an incision through the scarred tissue, outward turning of the lid and fixation with sutures. Generally, this will correct the trichiasis in most cases, although in more severe cases this might be insufficient. Post‐operative trichiasis can occur either through inadequate surgery or progressive scarring disease.

### Why it is important to do this review

Evidence from case series and randomised controlled trials suggests that upper lid surgery can be successful at abolishing trichiasis; however, typically 20% to 40% of eyelids suffer from post‐operative trichiasis by one year (Bog 1993; Bowman 2000; Burton 2005b; Rajak 2011b; Reacher 1990; Reacher 1992; Ward 2005; West 2005); and up to 60% at three years (Khandekar 2001). Therefore, it is important to understand the determinants of a good treatment outcome and identify strategies that lead to this.

Risk factors associated with post‐operative trichiasis include the severity of pre‐operative trichiasis (Alemayehu 2004; Burton 2005a; Rajak 2013); chlamydial infection (Zhang 2004); inflammation of the tarsal conjunctiva (Burton 2005a; Burton 2005b; Ward 2005); bacterial infection (Burton 2005a); and left eyes (Merbs 2005; West 2005).

The choice of treatment will depend on factors such as available resources and expertise, location (opportunity for follow up) and how advanced the disease is. The WHO strategy for the control of blinding trachoma calls for lid surgery to be delivered by ophthalmic assistants as well as ophthalmologists (Habtamu 2011), because the numbers of ophthalmologists are insufficient to provide the service. Ophthalmic assistants are usually taught to perform only one type of operation so it is important to ensure that the operation they use is known to be effective.

Although surgery generally produces good results, in many settings only a minority of patients with trichiasis will attend for surgery (Bowman 2002; Courtright 1994; West 1994). Delivery of surgery in the community or non‐surgical management of trichiasis may be more acceptable than surgery in a conventional hospital setting (Bowman 2000; Graz 1999; Rajak 2011a).

## Objectives

To assess the effects of interventions for trachomatous trichiasis for people living in endemic settings.

## Methods

### Criteria for considering studies for this review

#### Types of studies

We included randomised controlled trials of interventions for trachomatous trichiasis. The unit of randomisation was individuals or clusters, depending on the design of the study.

#### Types of participants

Participants in the trials were people with trachomatous trichiasis, defined as one or more eye lashes touching the globe when looking straight ahead.

#### Types of interventions

We included trials in which any intervention intended to prevent corneal opacification from prolonged lash‐globe contact was compared to another intervention or to no treatment. Surgical interventions were procedures to correct entropion or ablate the lash roots and non‐surgical interventions were taping of the lid margin or manual removal of the eyelashes (epilation). We included trials that compared:

- different surgical or non‐surgical interventions;
- medication to reduce post‐operative trichiasis;
- any intervention delivered in a hospital setting to the same intervention in a community setting;
- the same intervention delivered by different types of health care professionals.

#### Types of outcome measures

##### Primary outcomes

The primary outcome measure for this review was post‐operative trichiasis. This dichotomous outcome was defined as any lash touching the globe in the primary position. The critical points for follow‐up were three months, one year, and two years after treatment.

##### Secondary outcomes

Secondary measures were:

###### Visual acuity change

Measured by Snellen or logMAR visual acuity charts at one year and two years after treatment.

###### Corneal opacification change

Measured by clinical examination or photographic comparisons at one year and two years after treatment.

###### Acceptance of treatment

As measured by uptake/attendance for treatment.

###### Adverse effects

Any adverse effects, whether minor or severe, were recorded.

###### Quality of life

Any qualitative measures of discomfort/patient satisfaction were noted.

###### Economic evaluation

Where any cost data for interventions were available this was noted and commented on in the context of cost‐effectiveness. No formal cost‐effectiveness evaluation was planned.

### Search methods for identification of studies

#### Electronic searches

We searched CENTRAL (which contains the Cochrane Eyes and Vision Group Trials Register) (2015, Issue 4), Ovid MEDLINE, Ovid MEDLINE In‐Process and Other Non‐Indexed Citations, Ovid MEDLINE Daily, Ovid OLDMEDLINE (January 1946 to May 2015), EMBASE (January 1980 to May 2015), the ISRCTN registry (www.isrctn.com/editAdvancedSearch), ClinicalTrials.gov (www.clinicaltrials.gov) and the World Health Organization (WHO) International Clinical Trials Registry Platform (ICTRP) (www.who.int/ictrp/search/en). We did not use any date or language restrictions in the electronic searches for trials. We last searched the electronic databases on 7 May 2015.

See: Appendices for details of search strategies for CENTRAL (Appendix 1), MEDLINE (Appendix 2), EMBASE (Appendix 3), ISRCTN (Appendix 4), ClinicalTrials.gov (Appendix 5) and the ICTRP (Appendix 6).

#### Searching other resources

We contacted experts and researchers in the field to ask them for details of published, unpublished or ongoing trials. We searched the reference lists of relevant trials.

### Data collection and analysis

#### Selection of studies

Two review authors assessed the titles and abstracts resulting from the searches and selected all titles that referred to treatment for trachomatous trichiasis. Copies of possibly relevant trials were obtained and independently assessed by three review authors according to the 'Criteria for considering studies for this review'. Trials meeting these criteria were also assessed for quality.

#### Data extraction and management

We recorded data from included studies in a table under the following headings:

- methods ‐ including randomisation, intention‐to‐treat analysis;
- participants ‐ including cluster or individual, country, number; losses to follow up;
- interventions ‐ including types of surgery or other intervention, setting of intervention (community or clinic);
- outcomes ‐ including definitions of success or failure, visual acuity change, adverse effects.

Authors independently extracted outcome data. One author entered data into RevMan 5, which was checked by a second author.

#### Assessment of risk of bias in included studies

Three review authors assessed trial quality according to methods set out in Chapter 8 of the *Cochrane Handbook for Systematic Reviews of Interventions* (Higgins 2011) using the Cochrane Eyes and Vision Group Review Development Guidelines. In particular, random sequence generation, allocation concealment, performance bias, detection bias (masking of outcome graders to initial treatment), attrition bias (adequacy of follow up) and reporting bias were assessed. Risk of bias was graded as "Low Risk", "High Risk" and "Unclear Risk", according to the guidelines in Section 8 of the Handbook. We resolved disagreements between the authors by discussion.

#### Measures of treatment effect

The treatment effects were measured by calculating the odds ratio and 95% confidence interval for these.

#### Unit of analysis issues

The unit of analysis was generally the individual patient, with the exception of one cluster randomised trial.

#### Dealing with missing data

Studies were assessed for missing data and whether missing data were "missing at random". In one instance, data were found to be systematically excluded from the analysis of the primary outcome in the original report (recurrence occurring by three months after surgery). As sufficient information was presented in the report it was possible to reintroduce these primary outcome events here.

#### Assessment of heterogeneity

Heterogeneity between studies was assessed for those investigating the use of post‐operative antibiotic. Heterogeneity was considered in several categories: clinical, methodological and statistical.

#### Assessment of reporting biases

The studies were assessed in terms of the completeness of data presented.

#### Data synthesis

The interventions tested were varied and there was considerable heterogeneity. We present a descriptive summary of the results rather than a single summary statistic. Where the unit of randomisation was a cluster rather than an individual, data were analysed by cluster. The principal outcome measure was post‐operative trichiasis, which was defined as any lash touching the globe in the primary position. Odds ratios were calculated for the different interventions. Visual acuity data were presented as dichotomous data**—**statistically significant improvement or no improvement from pre‐ to post‐intervention. Data on adverse effects and acceptance of surgery were also presented as dichotomous data. The exception to this was the three studies examining the effect of azithromycin on the outcome of surgery. A meta‐analysis was performed for the dichotomous outcome of post‐operative trichiasis using a Mantel‐Haenszel, random‐effects model. For trials where there were more than two arms these were considered separately in relation to the reference intervention where appropriate, such as different surgical options. However, if two arms utilised the same intervention, they were combined.

#### Subgroup analysis and investigation of heterogeneity

No sub‐group analysis was performed.

#### Sensitivity analysis

No sensitivity analysis was performed.

## Results

### Description of studies

#### Results of the search

The search of electronic databases revealed a total of 656 reports. We retrieved nine papers for further assessment. All of these were randomised trials of interventions for trachoma trichiasis (Adamu 2002; Alemayehu 2004; Bowman 2000; Burton 2005a; Dhaliwal 2005; Graz 1999; Reacher 1990; Reacher 1992; West 2006).

An update search run in May 2015 identified 654 new records (Figure). The Trials Search Co‐ordinator removed 190 duplicate records, screened 464 records and removed 280 references that were not relevant to the scope of the review. We screened the remaining 184 references and discarded 176 reports as not relevant. We obtained eight full‐text reports of trials for assessment. We included four reports of four new studies (Gower 2013; Rajak 2011a; Rajak 2011b; Zhang 2006); and added a new report to Burton 2005a. Previously a study by West 2006 was awaiting assessment: this study has now been included and three new reports of the trial have been added to the review. In the previous version of this review Dhaliwal 2005 was excluded; however we have re‐assessed this study and have now included it in the review.

**1:**
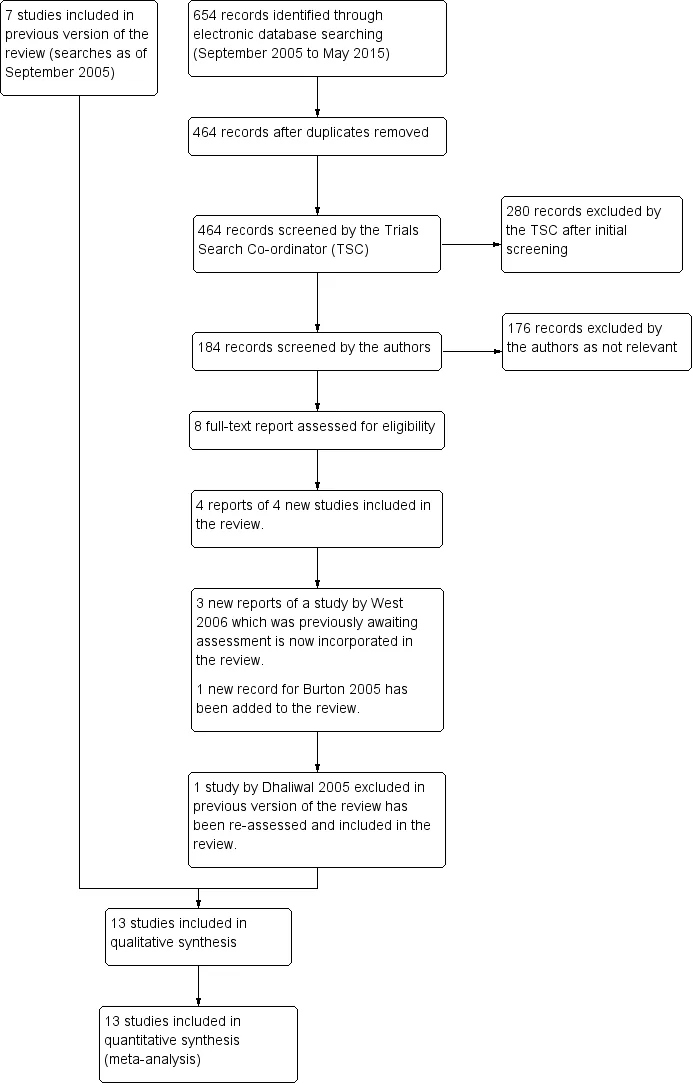
Study flow diagram.

#### Included studies

In some of the included studies, participants with bilateral disease were randomised by eye rather than by individual participant (details below); these studies were included on the basis that the majority of participants were randomised individually. Details of the included studies can be found in the 'Characteristics of included studies' table.

For ease of interpretation, studies have been grouped according to the aspect of trichiasis management they address:

- Surgical technique: Adamu 2002, Dhaliwal 2005, Gower 2013, Reacher 1990 and Reacher 1992 compared different surgical interventions.
- Epilation: Rajak 2011a compared epilation to surgery
- Lid taping: Graz 1999 examined lid taping versus epilation for managing trichiasis.
- Antibiotic treatment: Burton 2005a, West 2006 and Zhang 2006 examined whether peri‐operative azithromycin treatment reduced post‐operative trichiasis versus another or no treatment.
- Alternative suture materials: Rajak 2011b compared absorbable to non‐absorbable sutures.
- Surgery setting: Bowman 2000 investigated alternative settings for conducting surgery (health centre versus village).
- Personnel performing surgery: Alemayehu 2004 compared the outcome of surgery performed by different types of health care personnel.

##### Types of interventions

###### Surgical technique

Five studies compared different surgical interventions for trichiasis: Adamu 2002; Dhaliwal 2005; Gower 2013; Reacher 1990 and Reacher 1992.

Reacher 1990 randomly allocated individuals with major trichiasis (six or more lashes touching) to one of five operations: (1) bilamellar tarsal rotation; (2) tarsal advance and rotation; (3) eversion splinting; (4) tarsal advance; or (5) tarsal grooving. Minor trichiasis cases were excluded. Eyes with defective lid closure were also excluded from the study and all received a tarsal advance procedure. Surgery was performed by one of three surgeons.

In the Reacher 1992 study participants were again grouped (stratified) according to severity and then randomly allocated:

- Minor trichiasis **–** allocated to (1) electrolysis; (2) cryotherapy; or (3) bilamellar tarsal rotation.
- Major trichiasis **–** allocation to either (1) bilamellar tarsal rotation; or (2) tarsal advance and rotation.
- Defective lid closure **–** allocation to (1) tarsal advance and rotation; or (2) tarsal advance with buccal mucosal membrane graft.

Adamu 2002 compared bilamellar tarsal rotation and posterior lamellar tarsal rotation in patients with minor trichiasis, major trichiasis or defective lid closure with trichiasis. Surgery was performed by second‐year ophthalmic residents according to standardised procedures.

Dhaliwal 2005 compared three operations: (1) terminal tarsal rotation (a variant of the posterior tarsal rotation); (2) tarsal advance and rotation; and (3) tarsal grooving. One surgeon performed all of the procedures.

Gower 2013 compared standard bilamellar tarsal rotation surgery to the same procedure performed using an eyelid clamp. Surgery was performed by 18 surgical technicians, who were trained and certified to perform only one form of the operation. Patients were randomly allocated to the surgeon.

###### Epilation

Rajak 2011a investigated whether epilation was non‐inferior to posterior lamellar rotation surgery for minor trichiasis. Patients with minor trichiasis were randomised to either immediate surgery or repeated epilation with manufactured epilating forceps.

###### Lid taping

Graz 1999 compared manual removal of eyelashes (epilation) with the use of a double‐sided sticking plaster to force eyelashes away from contact with the globe: both interventions were undertaken prior to lid surgery. There were three groups: (1) epilation alone; (2) sticking plaster alone; and (3) sticking plaster for eight weeks then crossover to epilation.

###### Antibiotic treatment

Three separate trials have examined whether post‐operative oral azithromycin can reduce post‐operative trichiasis in three different countries: (1) The Gambia (Burton 2005a; (2) Ethiopia (West 2006); and (3) Nepal (Zhang 2006).

In the Gambian study all participants underwent posterior lamellar tarsal rotation and were prescribed tetracycline eye ointment twice daily for two weeks (Burton 2005a). Those randomised to the intervention group received a 1 g dose of azithromycin at the time of surgery; adults and children in these households were also given a single dose of azithromycin (adults 1 g and children 20 mg/kg) to reduce the risk of re‐infection. This medication was re‐administered at six months.

In the Ethiopian study, participants all underwent bilamellar tarsal rotation surgery (West 2006). They were then randomised to one of three intervention arms: (1) 1g dose of azithromycin for the patient alone; (2) 1 g dose of azithromycin for the patient and single‐dose azithromycin treatment for household members; (3) topical tetracycline (twice per day for six weeks).

In the Nepal study, participants all received bilamellar tarsal rotation surgery (Zhang 2006). At the end of surgery they were alternately given either azithromycin or a placebo.

###### Alternative suture materials

Rajak 2011b compared posterior lamellar rotation performed with silk sutures to the same procedure performed with absorbable polyglactin‐910 sutures.

###### Surgery setting

Bowman 2000 compared providing surgery in the participants' own village to surgery provided at the nearest health centre. Posterior lamellar tarsal rotation surgery was performed on all participants by one of five trained nurses or an ophthalmic assistant.

###### Personnel performing surgery

Alemayehu 2004 compared post‐operative trichiasis rates following surgery by ophthalmologists and non‐ophthalmologist integrated eye care workers (IECW) in Ethiopia. Subjects with trachomatous trichiasis (TT) were randomised to surgery by either an ophthalmologist or an IECW. Both groups used the bilamellar tarsal rotation procedure.

##### Types of participants

###### Surgical technique

Participants in the Reacher 1990 study were 165 adult Omanis with TT. Participants were grouped according to severity: minor trichiasis, major trichiasis or defective lid closure as defined above. Only those with major trichiasis were eligible for randomisation. Participants who had undergone previous treatment were included.

Reacher 1992 recruited 367 individuals diagnosed with TT by the Oman Prevention of Blindness Program. Trichiasis was graded as for the Reacher 1990 paper, and participants were grouped as minor trichiasis, major trichiasis or defective lid closure. Participants who had undergone previous treatment were included.

In the study by Adamu 2002, participants were 153 consecutive patients with TT presenting at a teaching hospital in Addis Ababa. Eight children (< 15 years) were included. All participants were graded pre‐operatively as having minor trichiasis, major trichiasis or defective lid closure. Previously operated eyes were excluded from the study.

Dhaliwal 2005 recruited 77 consecutive patients (90 eyes) in an eye clinic in India.

In Gower 2013, which was conducted in Tanzania, 1917 participants were enrolled through screening campaigns. Participants were at least 18 years old, had previously unoperated trichiasis and did not plan to move within 2 years. All degrees of trichiasis severity were eligible.

###### Epilation

Rajak 2011a was conducted in Ethiopia. Only adults (> 18 years) with minor trichiasis were eligible. 1300 individuals were identified and recruited through community outreach campaigns; 66% were female. At baseline by chance there was slightly more corneal disease in the epilation arm.

###### Lid taping

Graz 1999 randomised a total of 57 consecutive adult patients attending a hospital clinic: n = 21 randomised to sticking tape; n = 18 to epilation; n = 18 to sticking tape followed by epilation. Baseline characteristics were comparable except that five lids (number of participants not stated) in the sticking tape group had trichiasis due to a cause other than trachoma.

###### Antibiotic treatment

In Burton 2005a 451 participants with major trichiasis were enrolled; 70% were female. Baseline characteristics were similar between the two randomisation groups for age, ethnicity and severity of trichiasis.

In West 2006 1452 individuals with any degree of TT were recruited; 77% were female. The three arms were balanced in terms of baseline characteristics.

Zhang 2006 randomised 109 individuals with TT (both major and minor trichiasis); 73% were female. The baseline characteristics of the two arms were comparable.

###### Alternative Suture Materials

Rajak 2011b recruited 1300 adults with major TT; 78% were female. They were identified through community outreach campaigns.

###### Surgery setting

Bowman 2000 selected five districts in The Gambia that were known to have high levels of trichiasis and where village‐based surgery previously had not been available. The districts were subdivided to form eight pairs of village clusters. Within each pair, one cluster of villages was randomised to village‐based surgery and the other cluster of villages to health centre‐based surgery. Screening was undertaken by trained ophthalmic nurses. Only participants with major trichiasis (at least five in‐turned lashes) were eligible for inclusion, in accordance with the Gambian national guidelines for surgery. Participants ineligible for village‐based surgery for medical reasons were excluded from the trial and referred for health centre‐based surgery. In all 158 individuals with major trichiasis were recruited.

###### Personnel performing surgery

Alemayehu 2004 recruited 982 people with TT; 77% were female and 3% were children. Baseline characteristics of the randomised groups are not described.

##### Types of outcomes

In all but one of the studies a successful outcome included the absence of post‐operative trichiasis; this is usually defined as no lashes touching the eye. Specific outcomes for each study are described below.

###### Surgical technique

In the Reacher 1990 study a successful outcome was defined as no post‐operative trichiasis (no lashes in contact with the globe after surgery) and complete gentle closure of eyelids. There was no pre‐defined outcome point, and in the group with major trichiasis follow‐up varied from 5 to 11 months.

Reacher 1992 defined a successful surgical outcome as no post‐operative trichiasis, no further epilation/surgery during follow up period, complete lid closure, no over‐correction of lid margin, acceptable appearance to patient and examiner, and no onset of phthisis. They also examined the effect on visual acuity and the complication rate. Follow‐up points were not defined in the methodology, but occurred (on average) at 9 and 21 months.

Adamu 2002 defined success as no eyelash‐eyeball contact, complete lid closure and no over‐ or under‐correction. Recurrence was defined as eyelash‐eyeball contact in all positions of gaze or inward rotation of the lid margin. Visual acuity was measured pre‐ and post‐operatively. Final follow up was planned at three months.

Dhaliwal 2005 used several outcome measures at six months: trichiasis and/or entropion recurrence, palpebral aperture, an acceptable cosmetic appearance.

In Gower 2013, the primary outcome was the presence of one or more of the following unfavourable outcomes: post‐operative trichiasis; eyelid contour abnormality; or granuloma formation. Participants were re‐examined at six weeks, 12 months and 24 months. Both eyes in bilateral cases were included in the analysis, with an appropriate adjustment.

###### Epilation

In Rajak 2011a, the primary outcome measure was "failure" which was defined as either (1) five or more eyelashes touching the globe; or (2) a history of surgery performed in the trial eye at any point during the two‐year follow‐up period (in the case of the surgical arm this would be repeat surgery). Participants were re‐examined every six months for two years.

###### Lid taping

Graz 1999 collected data at 1, 4 and 12 weeks and recorded recurrence of trichiasis, visual acuity, patient discomfort and any adverse events.

###### Antibiotic treatment

Outcomes in the Burton 2005a study were assessed at 6 and 12 months post‐operatively. The primary outcome was post‐operative trichiasis. Secondary outcomes included visual acuity and patient perception of improvement by asking whether vision and pain was 'worse', 'same' or 'better'.

In West 2006 the primary outcome was post‐operative trichiasis. This was assessed at 2 weeks, 6 weeks, 6 months, and 12 months. Patients were examined again at 2 and 3 years.

Zhang 2006 evaluated three outcomes: post‐operative trichiasis; chlamydial infection; and active trachoma. Participants were re‐examined at 3, 6 and 12 months post‐operatively. If the participant developed "surgical failure", which was defined as five or more lashes touching the eye by three months, they were excluded from the analysis.

###### Alternative Suture Materials

Rajak 2011b The primary outcome measure was the proportion of those individuals seen at the 12‐month follow‐up who were found to have either (1) post‐operative trichiasis; or (2) a history of repeat TT surgery during the first year. Participants were re‐examined every six months for two years.

###### Surgery setting

There were three main outcomes in the Bowman 2000 study: (1) uptake of treatment in the local village compared to the health centre; (2) post‐operative trichiasis); (3) complication rate. Other parameters measured included time taken by the patient to travel to the operating room and cost implications associated with presenting for surgery.

###### Personnel performing surgery

The first follow‐up in the Alemayehu 2004 study was at seven days post‐operatively: those with post‐operative trichiasis at this assessment were deemed surgical failures and were excluded from the primary analysis; the main outcome was post‐operative trichiasis rate at three and six months. Those with post‐operative trichiasis at three months were excluded from the six‐month assessment. Analysis was by randomised group and also by presenting severity.

### Risk of bias in included studies

There are potential biases in several of the studies. These are considered under the following headings: allocation, blinding, incomplete data, and selective reporting. See Figure; Figure.

**2:**
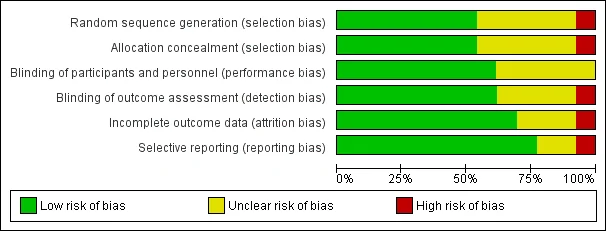
Risk of bias graph: review authors' judgements about each risk of bias item presented as percentages across all included studies.

**3:**
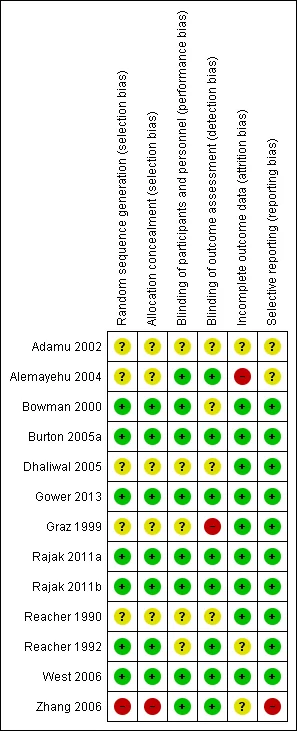
Risk of bias summary: review authors' judgements about each risk of bias item for each included study.

#### Allocation

##### Surgical technique

Adamu 2002 used an unspecified 'Lottery method' for randomisation. In bilateral cases the left eye always received the opposite treatment to that randomly allocated to the right eye, with no analytic adjustment for bilateral cases reported. The risk of bias is unclear given the limited details reported. In Gower 2013 randomisation assignments were created using permuted block sizes of 6 to 12 and placed in opaque envelopes; hence, the risk of bias is expected to be low. Reacher 1990 randomly allocated procedures using random number tables. Limited information is provided on the process and no details are provided on concealment; thus, the risk of bias is unclear. In the Reacher 1992 study, participants were randomly allocated using a computer‐generated sequence; assignments were concealed in opaque envelopes. Risk of bias is expected to be low. Dhaliwal 2005 randomised the study eye by a computer‐generated sequence. However, if a second eye was operated one of the other two options was used. The details are limited and, therefore, the risk of allocation bias is unclear.

##### Epilation

In Rajak 2011a participants were allocated to arm by a computer‐generated randomisation sequence and assignments were kept in sealed, opaque envelopes, leading to a low risk of bias.

##### Lid Taping

Graz 1999 provide no description of the randomisation process.

##### Antibiotic treatment

In Burton 2005a separate randomisation sequences were generated for each surgeon using random number tables and blocked in groups of four, and assignments were kept in sequential sealed envelopes, leading to a low risk of allocation bias. In West 2006, participants were randomly assigned to one of three intervention arms using a computer‐generated sequence with a variable block size, and assignments were kept in opaque containers until needed, leading to a low risk of bias. In Zhang 2006 participants all received bilamellar tarsal rotation surgery, and at the end of surgery they were alternately given either azithromycin or a placebo. This randomisation method makes the next assignment predictable, and hence makes the risk of allocation bias high.

##### Alternative Suture Materials

In Rajak 2011b the risk of allocation bias is expected to be low since people were allocated to arm by a computer‐generated randomisation sequence and assignments were maintained in opaque envelopes.

##### Surgery setting

In Bowman 2000, a cluster randomised trial, the communities were paired and randomly allocated before the numbers of TT patients was known, so risk of allocation bias low.

##### Personnel performing surgery

Alemayehu 2004 do not provide any details of the randomisation or concealment methods employed; hence, risk of bias is unknown.

#### Blinding

##### Surgical technique

Adamu 2002 provides no description of methods to mask either the participants or those performing the follow‐up examinations. In Gower 2013 randomisation assignments were placed in opaque envelopes and stored in a locked office until distribution and outcomes were assessed by a masked examiner. Reacher 1990 provides no information on the concealment of treatment allocation sequences. The follow‐up examinations were by a single observer, but it is unclear if he was masked to the allocation. In Reacher 1992 the allocation sequence was concealed in opaque envelopes. Follow‐up examinations were performed by a single masked observer. Dhaliwal 2005 provide no details about masking of the allocation prior to administration or during follow‐up; it is unclear if the observations were made by the same clinician who performed surgery or by a separate clinician. Therefore the risk of bias is unclear.

##### Epilation

In Rajak 2011a the randomisation sequence was concealed in opaque envelopes. Observers were independent of, and masked to, the allocation.

##### Lid taping

In Graz 1999 masking of the observers was not possible.

##### Antibiotic treatment

In Burton 2005a allocation was contained in opaque sequentially‐numbered envelopes and administered immediately following surgery by a nurse not involved in surgery or follow‐up assessment. Pre‐operative assessments and the 12‐month follow‐up assessments were made by the same observer. Assessments at six months were by a different observer. Both observers were masked to treatment allocation. Surgery was performed prior to the randomisation. In West 2006 the random treatment allocation was concealed in sequential opaque envelopes. In Zhang 2006 participants and observers were masked to whether they had received azithromycin or a placebo; however, this was on an alternating basis, rather than random allocation.

##### Alternative suture materials

In Rajak 2011b the randomisation sequence was concealed in opaque envelopes. Observers were independent of, and masked to, the allocation.

##### Surgery setting

In Bowman 2000 it would have been impossible to mask the patients or personnel to the location of surgery.

##### Personnel performing surgery

In Alemayehu 2004 the patients and the surgeons were not masked to the allocation. The follow‐up observations were made by ophthalmologists masked to the allocation.

#### Incomplete outcome data

##### Surgical technique

The studies by Adamu 2002, Gower 2013, Dhaliwal 2005, Reacher 1990 and Reacher 1992 all present a complete data set with high rates of follow‐up.

##### Epilation

Rajak 2011a presents a complete data set with high rates of follow‐up.

##### Lid taping

Graz 1999 presents a complete data set with high rates of follow‐up.

##### Antibiotic treatment

Burton 2005a presents a complete data set with high follow‐up. A geographically defined sub‐set of the original trial subjects was followed up to 4 years, with 94% follow‐up of those alive at 4 years. West 2006 presents a complete data set with high follow‐up. Similarly Zhang 2006 reports a complete data set with high follow‐up rate.

##### Alternative Suture Materials

Rajak 2011b presents a complete data set with high rates of follow‐up.

##### Surgery setting

Bowman 2000 presents complete outcome data.

##### Personnel performing surgery

Alemayehu 2004 had moderate loss to follow‐up. No comparative data on those lost to follow‐up are provided to determine risk of attrition bias.

#### Selective reporting

##### Surgical technique

Adamu 2002 provides limited information of their analytical protocol; therefore, risk of bias is unclear. The studies by Gower 2013, Reacher 1990 and Reacher 1992 all report a clear analytical approach which was followed in the results.

##### Epilation

The protocol for Rajak 2011a is published online and was followed.

##### Lid taping

Graz 1999 reports a clear analytical approach which was followed in the results.

##### Antibiotic treatment

Burton 2005a reports a clear analytical approach which was followed in the results. The protocol for West 2006 is published separately. Zhang 2006 excluded "surgical failures" from the analysis, defined as five or more lashes touching the globe at 3 months.

##### Alternative suture materials

The protocol for Rajak 2011b is published online and was followed.

##### Surgery setting

Bowman 2000 reports a clear analytical approach.

##### Personnel performing surgery

Alemayehu 2004 provides limited information on the analytical protocol; however, all participants seen at follow‐up were included in the analyses.

#### Other potential sources of bias

None identified.

### Effects of interventions

#### Surgical interventions

##### 1. Bilamellar tarsal rotation surgery compared to tarsal advance and rotation

Two studies reported this comparison. In Reacher 1990 the mean months of follow‐up per group ranged from 7.4 to 8.8 months (total range 5 to 11 months); in Reacher 1992 this was reported at 9 and 21 months after surgery.

###### 1.1 Post‐operative trichiasis

Eyes receiving bilamellar tarsal rotation surgery had lower odds of post‐operative trichiasis compared to eyes receiving tarsal advance and rotation (OR 0.29, 95% CI 0.16 to 0.50, 260 eyes, I² = 0%) (Analysis 1.1).

###### 1.2 Visual acuity change

Visual acuity was not reported in Reacher 1990.

In Reacher 1992 eyes receiving surgery for major trichiasis on average had half a line improvement in Snellen acuity (P < 0.001) but the difference between the intervention groups (if any) was not clearly reported.

###### 1.3 Corneal opacification change

Not reported.

###### 1.4 Acceptance of treatment

This was not reported in Reacher 1990.

In Reacher 1992 38 people refused their random allocation but it was not reported which groups they were allocated to.

###### 1.5 Adverse effects

Bilamellar tarsal rotation surgery was associated with more overcorrection of the entropion but the estimate was imprecise with very wide confidence intervals compatible with no effect, or more overcorrection in the tarsal advance group (OR 2.57, 95% CI 0.28 to 23.25, 312 eyes, I² = 0%) (Analysis 1.2). There were only 3 cases of overcorrection in the two trials but they were all in the bilamellar group.

Defective lid closure was also more common in the bilamellar group but again occurred rarely and the estimate of effect was very imprecise (OR 1.90, 95% CI 0.29 to 12.37, 312 eyes, I² = 0%). (Analysis 1.3).

###### 1.6 Quality of life

Not reported.

##### 2. Bilamellar tarsal rotation compared to techniques that do not create a full‐thickness incision of the tarsal plate and complete rotation of the lash‐bearing tissue

Only one study reported these comparisons (Reacher 1990). The mean months of follow‐up per group ranged from 7.4 to 8.8 months (total range 5 to 11 months)

###### 2.1 Post‐operative trichiasis

Bilamellar tarsal rotation was more effective than techniques that do not create a full‐thickness incision of the tarsal plate and complete rotation of the lash‐bearing tissue such as tarsal grooving and eversion splinting, and a non‐significant trend in the comparison with the tarsal advance procedure (Table).

**1:** Bilamellar tarsal rotation compared to techniques that do not create a full‐thickness incision of the tarsal plate and complete rotation of the lash‐bearing tissue

**Outcome**	**Other technique** **Follow‐up range 5 to 11 months**	**Bilamellar tarsal rotation** **n/N**	**Other technique** **n/N**	**Odds ratio (95% CI)**
Post‐operative trichiasis	Tarsal grooving	10/39	29/33	0.05 (0.01, 0.17)
	Eversion splinting	10/39	14/21	0.17 (0.05, 0.55)
	Tarsal advance	10/39	17/38	0.43 (0.16, 1.11)

Data from Reacher 1990. n = number of eyes with outcome; N = total number of eyes followed up

None of the other review outcomes were reported.

##### 3. Bilamellar tarsal rotation surgery compared to posterior lamellar tarsal rotation

One study reported this comparison (Adamu 2002). 256 upper eyelids of 153 people with trichiasis were randomly allocated to bilamellar tarsal rotation (n = 124) or posterior lamellar tarsal rotation (n = 132) and followed up to 3 months.

###### 3.1 Post‐operative trichiasis

After three months there was less post‐operative trichiasis in the bilamellar tarsal rotation group but the confidence intervals were wide and compatible with no effect, or more trichiasis (Table).

**2:** Bilamellar tarsal rotation surgery compared to posterior lamellar tarsal rotation

**Outcome**	**Follow‐up**	**Bilamellar tarsal rotation** **n/N**	**Posterior lamellar tarsal rotation** **n/N**	**Odds ratio** **(95% CI)**
Post‐operative trichiasis	3 months	12/124	15/132	0.84 (0.37, 1.86)

Data from Adamu 2002 n = number of eyes with outcome; N = total number of eyes followed up

###### 3.2 Visual acuity change

The authors stated that there was an improvement in vision after surgery but the data were not reported and it was of borderline statistical significance (P = 0.0515).

###### 3.3 Corneal opacification change

Not reported.

###### 3.4 Acceptance of treatment

Not reported.

###### 3.5 Adverse events

Lid‐notching and pyogenic granuloma were more common in the bilamellar than the posterior lamellar tarsal rotation operations (Chi² 9.54, P = 0.002) but no data were reported.

###### 3.6 Quality of life

Not reported.

##### 4. Terminal tarsal rotation compared to tarsal advance and rotation and tarsal grooving

One study reported these comparisons (Dhaliwal 2005). The study randomised 77 participants (90 eyes); all 77 participants had six‐month follow‐up data. Pre‐operatively entropion was found to be moderate in 65 eyes and severe in 25 eyes (although these terms were not defined). No eyes had defective lid closure. Bilateral TT was present in 13 people.

###### 4.1 Post‐operative trichiasis

After six months there was no significant difference in the rate of post‐operative trichiasis between the three procedures; however, the sample size in each group was insufficient to detect a statistically significant difference at a meaningful level.

###### 4.2 Visual acuity change

Not reported.

###### 4.3 Corneal opacification change

Not reported.

###### 4.4 Acceptance of treatment

Not reported.

###### 4.5 Adverse effects

Lid‐notching: (1) terminal tarsal rotation 9 (30%); (2) tarsal advance and rotation 6 (20%); and (3) tarsal grooving 10 (33%)

Pyogenic granuloma: (1) terminal tarsal rotation 3 (10%); (2) tarsal advance and rotation 5 (17%); and (3) tarsal grooving 3 (10%)

###### 4.6 Quality of life

There was no difference between the three groups in terms of the proportion of patients who were satisfied with the cosmetic appearance following surgery: (1) terminal tarsal rotation 28 (93%); (2) tarsal advance and rotation 27 (90%); and (3) tarsal grooving 27 (90%).

##### 5. Bilamellar tarsal rotation with a clamp compared to bilamellar tarsal rotation without a clamp

One study (Gower 2013) reported this comparison. The study randomised 1917 participants (3345 eyes) and followed up to two years.

###### 5.1 Post‐operative trichiasis

There was more post‐operative trichiasis in the clamp surgery group at two years. After adjustment for correlation between eyes and for surgeon, age, sex and baseline TT severity the adjusted OR was 1.36, 95% CI 0.96 to 1.93. The lower confidence interval includes, but is fairly close to 1 (no effect).

###### 5.2 Visual acuity change

Not reported.

###### 5.3 Corneal opacification change

Not reported.

###### 5.4 Acceptance of treatment

Not reported.

###### 5.5 Adverse effects

Eyelid contour abnormalities were less frequent in the clamp surgery group (adjusted OR 0.65, 95% CI 0.44 to 0.98, 3343 eyes).

Granulomas were less frequent in the clamp surgery group (adjusted OR 0.67, 95% CI 0.46 to 0.97, 3343 eyes).

###### 5.6 Quality of life

Not reported.

##### 6. Bilamellar tarsal rotation surgery compared to cryotherapy or electrolysis

###### 6.1 Post‐operative trichiasis

Post‐operative trichiasis in 166 eyes with minor trichiasis was reported by Reacher 1992; follow‐up ranged from 1 to 21 months.

Bilamellar tarsal rotation was more effective than destruction of the lashes by cryotherapy or electrolysis (Table).

**3:** Bilamellar tarsal rotation surgery compared to cryotherapy or electrolysis

**Outcome**	**Other technique** **Follow‐up range 1 to 21 months**	**Bilamellar tarsal rotation** **n/N**	**Other technique** **n/N**	**Odds ratio (95% CI)**
Post‐operative trichiasis	Cryotherapy	6/52	41/57	0.05 (0.02, 0.14)
	Electrolysis	6/52	30/57	0.12 (0.04, 0.32)

Data from Reacher 1992 n = number of eyes with outcome; N = total number of eyes followed up

###### 6.2 Visual acuity change

Treatment of minor trichiasis was not associated with any improvement in vision but the differential effect of treatment, if any, was not clearly reported.

###### 6.3 Corneal opacification change

Not reported.

###### 6.4 Acceptance of treatment

Not reported.

###### 6.5 Adverse effects

Not reported.

###### 6.6 Quality of life

Not reported.

##### 7. Posterior lamellar tarsal rotation surgery compared to epilation

One trial reported this comparison (Rajak 2011a). At baseline 1300 participants with minor trichiasis were randomised to surgery or epilation and followed up for two years.

###### 7.1 Post‐operative trichiasis

Trichiasis defined as one or more lashes touching the globe or repeat surgery was less frequent in the surgery group over two years (Table).

**4:** Posterior lamellar tarsal rotation surgery compared to epilation

**Outcome**	**Follow‐up**	**Posterior lamellar tarsal rotation surgery** **n/N**	**Epilation** **n/N**	**Odds ratio (95% CI)**
Post‐operative trichiasis	Two years	114/637	298/641	0.25 (0.19, 0.32)
Visual acuity change: deterioration of one or more lines of visual acuity	One year	168/620	174/598	0.91 (0.71, 1.16)
	Two years	207/613	224/603	0.86 (0.68, 1.09)
Deterioration in corneal opacification	One year	7/620	12/598	0.56 (0.22, 1.43)
	Two years	25/613	33/603	0.73 (0.43, 1.25)

Data from Rajak 2011a. n = number of eyes with outcome; N = total number of eyes followed up

###### 7.2 Visual acuity change

There was no evidence of a difference between the two groups in deterioration in visual acuity between baseline and one year or two years; however the confidence intervals include 1, therefore they are consistent with no effect (Table).

###### 7.3 Corneal opacification change

Progression in corneal opacification was infrequent in both groups at one and two years. There was a non‐significant trend to less deterioration in corneal opacification between baseline and one or two years in the surgery group; however the wide confidence intervals mean we cannot exclude the possibility that there is less deterioration in the epilation group or no difference between procedures (Table).

###### 7.4 Acceptance of treatment

All participants accepted their initial randomisation allocation. At two years 185/593 (31%) of individuals who were still epilating accepted the offer of free community‐based surgery.

###### 7.5 Adverse effects

In the surgery arm, by two years, 105 (16%) of eyes had developed recurrent trichiasis, granulomas developed in 18 (2.9%) and eyelid contour abnormalities were reported in 29 (4.7%).

###### 7.6 Quality of life

At 12 months 43% of participants in the surgery arm recalled "severe" treatment pain compared to 27% of people in the epilation group. More people in the surgery groups reported better subjective improvement in vision at 12 months (78%) compared to 33% of the epilation group, and less eye pain (44% compared to 70%), and eye watering (51% compared to 63%). All these differences were statistically significant.

##### 8. Absorbable (polyglactin‐910) compared to non‐absorbable (silk) sutures

One trial investigated this comparison (Rajak 2011b). 1300 participants with major trichiasis were randomised to absorbable polyglactin‐910 sutures or silk sutures and followed‐up at one year and two years.

###### 8.1 Post‐operative trichiasis

Post‐operative trichiasis at one year and two years was similar between the two groups (Table).

**5:** Absorbable (polyglactin‐910) compared to non‐absorbable (silk) sutures

**Outcome**	**Follow‐up**	**Absorbable sutures** **n/N**	**Silk sutures** **n/N**	**Odds ratio (95% CI)**
Post‐operative trichiasis	One year	114/608	120/628	0.98 (0.73, 1.30))
	Two years	117/609	118/609	0.99 (0.75, 1.32)
Visual acuity change: deterioration of one or more lines	One year	167/608	146/628	1.25 (0.97, 1.62)
	Two years	191/609	198/609	0.95 (0.75, 1.21)
Deterioration in corneal opacification	One year	6/608	11/628	0.56 (0.21, 1.52)
	Two years	24/609	18/609	1.35 (0.72, 2.51)

Data from Rajak 2011b. n = number of eyes with outcome; N = total number of eyes followed up

###### 8.2 Visual acuity change

Change (deterioration of one or more lines) in visual acuity between baseline and two years was similar in the two groups (Table).

###### 8.3 Corneal opacification change

There was less deterioration in corneal opacification between baseline and one year in the absorbable sutures group and more at two years but the confidence intervals were wide and compatible with no effect (Table).

###### 8.4 Acceptance of treatment

Not reported.

###### 8.5 Adverse effects

Fewer granulomas were found in the absorbable sutures group at six months (OR 0.63, 95% CI 0.40 to 0.99, 1187 eyes).

###### 8.6 Quality of life

Not reported.

#### Non‐surgical interventions

##### 9. Sticking tape compared to epilation

One study reported this comparison and followed up to 3 months (Graz 1999). The study randomised 57 participants.

###### 9.1 Post‐operative trichiasis

The use of sticking tape alone was significantly more effective at preventing eyelashes from touching the eye compared to epilation alone at three months (Table).

**6:** Sticking tape compared to epilation

**Outcome**	**Follow‐up**	**Sticking tape** **n/N**	**Epilation** **n/N**	**Odds ratio (95% CI)**
Post‐operative trichiasis	3 months	6/21	18/18	0.01 (0, 0.22)

Data from Graz 1999. n = number of eyes with outcome; N = total number of eyes followed up

###### 9.2 Visual acuity change

Snellen visual acuity was measured using the 'E' optotype, but outcomes were not reported.

###### 9.3 Corneal opacification change

Not reported.

###### 9.4 Acceptance of treatment

Attendance for treatment was not recorded but it was mentioned in the discussion that there may have been compliance issues.

###### 9.5 Adverse effects

Not reported.

###### 9.6 Quality of life

A patient questionnaire of six closed‐response questions was used to measure levels of discomfort; results were summarised as 'complaint' versus 'no complaint'. Patients found the less successful treatment of epilation significantly more uncomfortable than the sticking tape (P = 0.002); this was only reported on those with unilateral trichiasis.

##### 10. Azithromycin compared to no azithromycin

Three studies reported this comparison (Burton 2005a; West 2006; Zhang 2006).

Burton 2005a randomised 451 people with major trichiasis only to receive azithromycin or no azithromycin at time of surgery; household members were also treated.

West 2006 (STAR trial) randomised participants to three groups: azithromycin to patient only (483); azithromycin to patient and household (485); or tetracycline (484).

Zhang 2006 randomised 109 people (148 eyes) with either minor and major trichiasis to azithromycin or placebo at time of surgery.

###### 10.1 Post‐operative trichiasis

There was heterogeneity between the trials in terms of postoperative trichiasis. West 2006 found a lower recurrence with azithromycin, while Burton 2005a and Zhang 2006 did not find a significant difference. In combining the data from the three trials, overall there was less post‐operative trichiasis at one year in people given azithromycin; however the combined result effect was uncertain as the confidence intervals include 1 (OR 0.85, 95% CI 0.63 to 1.14, 1954 eyes, 3 studies) (Analysis 2.1).

At three years in the STAR trial (West 2006) there was less post‐operative trichiasis but again the overall effect was uncertain. A similar pattern was seen at four years follow‐up in Burton 2005a (Analysis 2.2).

###### 10.2 Visual acuity change

In Burton 2005a visual acuity improved in 57.6% of eyes by 12 months. There was an overall improvement of 0.14 logMAR (P < 0.0001). Data were not reported by randomised groups.

In the STAR trial (West 2006) a comparison of the change in visual acuity between baseline and six months on a consecutive subset of 439 study subjects did not find a significant difference in visual acuity outcomes between the azithromycin and control groups but data were not reported.

###### 10.3 Corneal opacification change

Not reported.

###### 10.4 Acceptance of treatment

In Burton 2005a all study participants accepted their allocated treatment.

In the STAR trial (West 2006) and Zhang 2006 this was not reported.

###### 10.5 Adverse effects

In Burton 2005a one participant had a post‐operative skin infection that settled on oral antibiotics. Two individuals had defective lid closure of less than 2 mm lagophthalmos.

In West 2006 there was a similar rate of adverse events in the azithromycin and control groups with a rate per 100 person of adverse events (death/illness/ocular) at 6 weeks of 2.90 (1.93‐4.19) in the azithromycin group and 3.10 (1.73‐5.11) in the tetracycline group.

Adverse effects were not reported in Zhang 2006.

###### 10.6 Quality of life

In Burton 2005a 77% reported improvement in vision and 94.3% felt the operated eye was more comfortable but this was not analysed by randomised groups.

In West 2006 trichiasis surgery had a marked benefit on physical functioning in a sub‐group analysis (Wolle 2011), but this was not analysed by groups allocated to azithromycin or control.

Quality of life was not reported in Zhang 2006.

#### Settings and personnel

##### 11. Community‐based compared to health centre‐based surgery

One study reported this comparison (Bowman 2000). This was a cluster‐randomised trial. Eight paired clusters of villages were randomised to community or village‐based surgery (86 participants) or health centre‐based surgery (72 participants).

###### 11.1 Post‐operative trichiasis

More people in the village‐based surgery group had post‐operative trichiasis at 3 months compared to the health centre‐based surgery group but the confidence intervals were wide and compatible with no effect or less trichiasis (OR 1.44, 95% CI 0.26 to 7.90).

###### 11.2 Visual acuity change

Not reported.

###### 11.3 Corneal opacification change

Not reported.

###### 11.4 Acceptance of treatment

In six of the eight pairs of clusters surgical uptake was higher for village‐based surgery; however, this difference might have occurred by chance (difference 20%, 95% CI −9% to 49%). Analysed by individual, 57/86 (66%) in the village‐based clusters attended for surgery compared to 32/72 (44%) in the health centre‐based group (OR 2.46, 95% CI 1.29 to 4.68).

###### 11.5 Adverse effects

A total of four events are reported but there was said to be no difference between groups (data not reported).

###### 11.6 Quality of life

Not reported.

###### 11.7 Cost

The cost of travel was significantly less in the clusters randomised to community‐based surgery (difference between means 10.5 Dalasi, 95% CI 6.07 to 14.93). Journey time to village‐based surgery was significantly less (difference between means 36 minutes, 95% CI 15.37 to 56.63).

##### 12. Ophthalmologist compared to integrated eye care worker (IECW)

One study reported this comparison (Alemayehu 2004). Of the 982 randomised, 713 (73%) attended the three‐month outcome assessment: 370/713 (52%) ophthalmologist group; 343/713 (48%) IECW group.

This study reported a linear trend for increased risk of recurrence with increasing severity of pre‐operative entropion (X² 22, P < 0.001). Randomisation was not stratified according to severity. Data on pre‐operative disease severity is not presented by the two randomisation groups. It is possible that either group may have treated a higher proportion of patients with severe disease, which could influence the outcome. A seven‐day post‐operative check was planned to identify surgical failures (which were then excluded from further analysis) but the outcomes from this assessment were not reported. If there was a significant difference in the number of failures between the groups at that stage, it would affect the interpretation of the results.

###### 12.1 Post‐operative trichiasis

There was more post‐operative trichiasis in the ophthalmologist group at 3 months but the confidence intervals were wide and compatible with no effect or less trichiasis (OR 1.32, 95% CI 0.83 to 2.11, 713 eyes).

There was a difference in the success rates of the two IECWs: one operated on 184 of whom 12 (6.5%) developed recurrence, the other operated on 159 of whom 22 (13.8%) developed recurrence (OR 2.3, 95% CI 1.1 to 4.8).

None of the other review outcomes were reported.

## Discussion

### Summary of main results

Trachomatous trichiasis is a significant ophthalmic public health problem in several regions of the world, particularly in sub‐Saharan Africa. The evidence identified in this review serves to highlight not only what is currently known about the effectiveness of treatment for this condition but also the challenges involved in delivering the necessary care and achieving good long‐term outcomes to prevent blindness.

#### Surgical interventions

Detailed descriptions of the different surgical procedures are provided elsewhere (Rajak 2012). Some evidence suggests that operations in which the full‐thickness of the tarsal plate is incised and the terminal lash‐bearing tarsus is rotated so that the lid margin is everted are more effective than procedures that do not involve this (Reacher 1990). It was possible to pool the results of Reacher 1990 and Reacher 1992, which found evidence that bilamellar tarsal rotation has lower post‐operative trichiasis rates than tarsal advance and rotation. This unilamellar procedure was performed by placing the sutures through the marginal strip of the tarsal plate and a second set from the upper end of the tarsal plate into the anterior lamella. In Adamu 2002 a variant of the bilamellar tarsal rotation was compared to the posterior lamellar tarsal rotation (also known as the Trabut procedure), in which the sutures were placed above the lashes, similar to the bilamellar technique. The same unilamellar technique as that used by Adamu 2002 was used in a case series in East Africa with similar anatomical success (Bog 1993). It is possible that the difference in results reflects the difference in technique, but the studies also had differing follow‐up periods (times of assessment), which may have influenced the outcomes.

At present both the bilamellar and posterior lamellar tarsal rotation operations are both extensively used in trachoma‐endemic countries. There is currently no conclusive evidence that bilamellar surgery is superior to the posterior lamellar operation. Although cases of overcorrection and exposure were more common following bilamellar lid surgery, the risk was very low and the difference was not statistically significant in any study. It appears that both bilamellar and posterior lamellar tarsal rotation procedures are safe operations. The only common complication was post‐operative trichiasis.

The use of a lid clamp in the bilamellar tarsal rotation procedure did not reduce post‐operative trichiasis but was associated with better eyelid contour outcomes and fewer granulomas (Gower 2013). Both lid rotation operations are relatively simple and require little equipment. Ophthalmologists, nurses, ophthalmology trainees and IECWs undertook the surgery in the included studies. It is possible that certain types of surgery are more effective than other types in specific situations. To our knowledge this has not yet been tested in any trial.

Interventions to treat minor trichiasis by destroying the lashes, such as cryotherapy and electrolysis, appear to have low success rates in preventing recurrent trichiasis compared to bilamellar tarsal rotation surgery (Reacher 1992). As the equipment required is costly and can be difficult to maintain, this strategy is not recommended.

#### Epilation

Epilation is widely practised in most regions that have a high prevalence of trichiasis and it may have a role in the management of minor trichiasis where there are only a few peripheral lashes and a patient declines surgery. The study that investigated this was a non‐inferiority trial comparing enhanced epilation (high‐quality forceps and training) to posterior lamellar tarsal rotation surgery for minor trichiasis cases only (Rajak 2011a). The primary endpoint in that trial was progression to major trichiasis with a prespecified non‐inferiority margin of 10%. The cumulative risk of failure was 13.2% in the epilation group and 2.2% in the surgical group, with a risk difference of 11% (95% CI 8.1% to 13.9%, which includes the 10% non‐inferiority margin). Therefore, the trial provided an inconclusive result relative to the predefined 10% non‐inferiority margin. The proportion having any degree of trichiasis during follow‐up was significantly higher in the epilation group. However, over a two‐year period the important secondary outcome measures of change in visual acuity and corneal opacity showed no significant difference. It is notable that at two years only 31% of those still epilating accepted the offer of surgery.

#### Lid‐taping

Double‐sided sticking plaster requires replacement of the plaster every week. As a temporary measure, however, the use of sticking plaster to evert the lashes is useful and in the study by Graz 1999 was superior to, and more comfortable than, epilation.

#### Antibiotic treatment

Post‐operative trichiasis was observed more often when the tarsal conjunctiva was inflamed (Burton 2005a; Burton 2005b; Rajak 2011b; West 2005). This clinically apparent inflammatory reaction could arise for a number of reasons: a smouldering immunologically driven process, infection with chlamydia or other bacteria, or mechanical irritation from the lashes (Burton 2010;Burton 2012). It is likely that chronic inflammation is the basis of progressive conjunctival scarring. Adjuvant therapy may reduce the risk of recurrent scarring and trichiasis.

Three studies with heterogeneous designs, populations and study participants have investigated whether azithromycin can reduce trichiasis recurrence. Azithromycin is a broad spectrum antibiotic with some anti‐inflammatory properties. The first study was conducted in a programmatic context where many different surgeons were operating in The Gambia, a country with a very low prevalence of chlamydial infection, and recruited only major trichiasis cases (Burton 2005a). This study did not find an effect from azithromycin at one or four years, and overall recurrence rates were relatively high with significant inter‐surgeon heterogeneity. The second (and largest) study, which included both major and minor trichiasis from an area of Ethiopia with high chlamydial infection rates and a limited number of surgeons, found a significantly lower trichiasis recurrence rate in the azithromycin arm at one year, but not at three years (West 2006). The third study, from Nepal, did not find an overall significant difference at one year (Zhang 2006). A sub‐group analysis suggested a possible effect for major trichiasis cumulatively to one year. None of the other sub‐groups showed a significant effect. It is not possible to draw a definite conclusion about the impact of azithromycin on post‐operative trichiasis due to the study heterogeneity. The pooled analysis did not find a significant reduction in post‐operative trichiasis. It appears that where post‐operative trichiasis rates are low, there may be some benefit of azithromycin treatment; however, under operational/programmatic conditions with higher overall post‐operative trichiasis rates there was no observed effect. Non‐chlamydial bacteria have been frequently cultured from the conjunctiva of people with trichiasis (Burton 2005a).

#### Alternative suture materials

The study comparing silk with absorbable sutures found no difference in the recurrence rates at one or two years (Rajak 2011b). In many settings patients with trichiasis have to travel long distances to obtain surgical services. The typical practice is to use silk sutures, which have to be removed a week to 10 days post‐operatively. This incurs additional transport and time for both the patient and surgical team, adding to the overall cost of surgery. However, in many settings absorbable sutures are substantially more expensive than silk sutures. Occasionally silk sutures are not removed, which can cause granuloma formation and serious complications for the cornea if left in long‐term. Therefore, the use of absorbable sutures may be preferable for cost reasons where these can be afforded; a formal cost benefit analysis is needed to answer this question.

#### Surgery setting

The uptake of trichiasis surgery is often low (Courtright 1994; West 1994); strategies that increase the proportion of patients who attend for surgery need to be developed. Bowman 2000 showed that it was less costly, and took less time, for participants to attend surgery in the community and that community‐based surgery was as safe and as effective as surgery in a health centre. However the uptake of surgery was only 20% better for village‐based surgery than for health centre‐based surgery (when analysed by cluster). It is not mentioned whether the resource implications for setting up village‐based surgery were greater than those required for health centre‐based surgery and it may be that this needs to be considered in the light of the reasonably small improvement in uptake. The village‐based approach to delivering surgery may work better in certain environments than others and should be considered along with other strategies for further research.

#### Personnel performing surgery

Most regions where trichiasis is prevalent have few ophthalmologists, and other health workers need to be trained to undertake the surgery in order to provide an adequate level of service. The evidence from the Alemayehu 2004 study suggests that surgery performed by a specially‐trained integrated eye worker is as successful as that undertaken by an ophthalmologist. Some caution needs to be applied in interpreting this result as the early recurrences (occurring by seven days) were excluded from the analysis. While this is very encouraging in terms of providing not only man‐power but also a high‐quality service, care needs to be taken to ensure appropriate specialist training is in place. Multiple studies have shown that success rates vary across surgeons (Burton 2005a; West 2005). This finding probably reflects varying levels of training, innate skill and experience, and serves to highlight the need not only for a high standard of basic training but also for ongoing monitoring and support.

#### Secondary outcome measures

Improvement in visual acuity or prevention of visual acuity deterioration is the primary long‐term aim of treating trachomatous trichiasis. However, this requires long‐term studies and therefore such evidence would be very difficult to obtain. The studies included here do not provide the opportunity to evaluate whether trichiasis surgery prevents long‐term loss of vision, because surgery has never been compared directly with no treatment. However, several studies have found modest but significant improvement in vision following surgery (Burton 2005a; Rajak 2011b; Reacher 1992; West 2006). Some of the visual impairment prior to surgery may be due to photophobia, lacrimation or irritation, or combinations of these three factors, caused by trichiatic lashes. Correction of the trichiasis should relieve these symptoms. Corneal scarring generally does not improve following surgery, although one study which compared corneal photographs before surgery to those at 2 years did find a degree of improvement in a minority of people (Rajak 2011b).

Some trials have reported relatively high rates of eyelid contour abnormalities, which were less frequent with the use of a lid clamp (Rajak 2011b, Gower 2013). Other factors that are likely to affect patient satisfaction with surgery are comfort and appearance. Despite a lack of evidence for improvement or preservation of vision, Bowman 2002 reported 85% of operated patients were pleased with the outcome of surgery and 94% would recommend the operation to others. In the Burton 2005a study, 94% of patients said they were more comfortable a year after surgery, compared to their pre‐operative condition. Careful study of these important subjective factors may help provide a more complete picture of successful treatment and maybe even identify motivators for attendance.

### Quality of the evidence

It is important to recognise the real difficulties of conducting studies in areas where resources and access to health care are limited, but there are several quality issues that must be highlighted in order for the studies to be interpreted appropriately. The grade of the evidence presented by many of the thirteen studies was high; however, there was some variability in the quality either due to small sample size or unclear or sub‐optimal methodology.

For several the sample size was relatively low and probably insufficient to address the question (Dhaliwal 2005; Graz 1999; Reacher 1990; Zhang 2006).

Most studies randomised participants; for several, however, sufficient information is not provided to determine the risk of bias associated with randomization (Adamu 2002; Alemayehu 2004; Graz 1999; Reacher 1990). In one study patients were assigned on an alternating basis (Zhang 2006), and this approach is vulnerable to bias because the assignment coordinator could adjust the enrolment order based on his/her preferences or beliefs.

Participant masking to intervention was not possible in most studies and would not be expected to influence the outcome, since typically an independent observer evaluated a non‐subjective outcome (presence of lashes touching the eye). Masking of outcome assessors is more crucial for unbiased reporting; however, in four studies it is unclear whether masking was done (Adamu 2002; Bowman 2000; Graz 1999; Reacher 1990). While this must be considered a quality concern, the reality may have been that researchers were working with a limited number of eye care professionals and did not have the resources to exclude some staff from delivery of care in order to ensure adequate masking.

The length of follow‐up in many of the included trials is quite short, limiting information regarding the long‐term effectiveness of the treatments. Long‐term follow‐up studies indicate that trichiasis continues to return after several years, but at a slower rate than during the first year post‐operatively (Rajak 2013; West 2006). With the exception of Reacher 1992, Burton 2005a, West 2006, Rajak 2011a, Rajak 2011b and Gower 2013, all studies had less than 12 months of follow‐up. Many areas where trachomatous trichiasis remains a significant public health problem are isolated and poor, and some of the population may be semi‐nomadic, making longer follow‐up difficult to achieve. The relatively short duration of follow‐up did, however, mean there were generally high levels of follow‐up.

## Authors' conclusions

**Implications for practice**

The evidence summarised in this review provides some indication of the basis for practice but one must remember that there are weaknesses in some of the data that may make the results unreliable.

There is less risk of recurrence if the full thickness of the tarsal plate is incised and the lash‐bearing tissue is rotated away from the globe to evert the lid margin compared to procedures that do not involve a full‐thickness tarsotomy. This can be achieved by either unilamellar or bilamellar lid surgery. Operations such as tarsal grooving, tarsal advance and eversion splinting are less effective. The bilamellar tarsal rotation is better than either cryotherapy or electrolysis in rendering a patient trichiasis‐free.

The optimal management of minor trichiasis remains uncertain. However, for patients with minor trichiasis who refuse surgery or where surgery is unavailable, epilation is a generally acceptable second‐line alternative treatment with comparable visual outcomes.

Post‐operative azithromycin may be associated with reduced risk of post‐operative trichiasis; however, this effect is less certain under programmatic conditions than under high‐quality surgery by a few highly‐skilled integrated eye care workers.

Silk and absorbable sutures have comparable outcomes. However, absorbable sutures have practical operational advantages in settings where they are affordable.

Local health workers (nurses, medical assistants or non‐ophthalmologist doctors) may be trained to a level where they can perform trichiasis surgery as effectively as an ophthalmologist; however, it is essential that local health workers receive good training, surgical certification and follow‐up supportive supervision in order to help ensure high‐quality surgery is maintained.

If uptake for trichiasis surgery is low in central settings, consideration should be given to providing it in patients' own communities.

**Implications for research**

***Recurrent trichiasis:*** the recurrence rate of trichiasis following surgery remains high in most programmatic settings, regardless of the intervention used. Recurrence is strongly associated with tarsal conjunctival inflammation, the cause of which is not well understood. Research is needed to improve understanding of the reasons why trichiasis recurs and to investigate targets for possible adjuvant therapy. Further trials are needed to compare the long‐term results of bilamellar and posterior lamellar tarsal rotation surgery, the two most commonly used operations. Since it is now known that the risk of recurrence is influenced by entropion severity, this must be taken into account in the design of future trials.

***Acceptability of treatment:*** a major obstacle to successful trichiasis surgery is failure to attend for an operation. Further research is required to identify other means of increasing the proportion of people who attend for surgery. It may be possible to identify the perceived benefits of lid surgery to the patient—such as free transport or improved cosmesis—and to use these perceived benefits to persuade others to attend.

***Pattern of surgical provision:*** we do not yet know the most effective way of delivering trichiasis surgery: is it through a high‐volume surgical camp, or through a single surgeon working long‐term in a specific district? Further studies are required to determine not only which method will give the best uptake, but also which is most cost effective and is associated with the lowest recurrence rate.

***Quality of surgery:*** there is a need for research to understand the determinants of a successful surgical training programme. There is a need to assess methods to audit the outcome of surgery by individual surgeons. This information then needs to be fed back to improve training programmes.

***Quality of life:*** although it has been shown that most trichiasis patients are happy with the results of their surgery the impact of surgery on quality of life and what might enhance this is a neglected area of research,

## What's new

**Table:** 

**Date**	**Event**	**Description**
14 December 2016	Amended	Amendment to Analysis 2.1

## History

Protocol first published: Issue 1, 2003 Review first published: Issue 3, 2006

**Table:** 

**Date**	**Event**	**Description**
15 December 2015	Amended	Minor amendment to Analysis 2:1: change from 'subtotals only' to 'no totals'
19 May 2015	New search has been performed	Issue 11, 2015: Electronic searches have been updated
19 May 2015	New citation required and conclusions have changed	Issue 11, 2015: 6 studies (4 new: Gower 2013; Rajak 2011a; Rajak 2011b; Zhang 2006 and 2 from previous searches: Dhaliwal 2005; West 2006) have been included in this update
29 October 2008	Amended	Converted to new review format.
1 March 2006	New citation required and conclusions have changed	Substantive amendment

## Appendices

### Appendix 1. CENTRAL search strategy

#1 MeSH descriptor: [Trachoma] explode all trees #2 MeSH descriptor: [Trichiasis] this term only #3 MeSH descriptor: [Chlamydia trachomatis] explode all trees #4 MeSH descriptor: [Eye] explode all trees #5 #3 and #4 #6 trachoma* or tracoma* or trichiasis #7 MeSH descriptor: [Entropion] explode all trees #8 entropion #9 #6 or #7 or #8 #10 #1 or #2 or #5 or #9 #11 MeSH descriptor: [Ophthalmologic Surgical Procedures] explode all trees #12 MeSH descriptor: [Eyelids] explode all trees #13 MeSH descriptor: [Eyelid Diseases] explode all trees #14 surg* or operat* or incis* #15 tars* near/3 rotat* #16 tars* near/3 plat* #17 tarsotom* or trabut or ballen or streatfield #18 epilat$ or electrolysis or cryo* #19 (tap* or plaster*) near (eye*) #20 (tap* or plaster*) near (lid*) #21 #11 or #12 or #13 or #14 or #15 or #16 or #17 or #18 or #19 or #20 #22 #10 and #21

### Appendix 2. MEDLINE (Ovid) search strategy

1. randomized controlled trial.pt. 2. (randomized or randomised).ab,ti. 3. placebo.ab,ti. 4. dt.fs. 5. randomly.ab,ti. 6. trial.ab,ti. 7. groups.ab,ti. 8. or/1‐7 9. exp animals/ 10. exp humans/ 11. 9 not (9 and 10) 12. 8 not 11 13. exp trachoma/ 14. trichiasis/ 15. exp chlamydia‐trachomatis/ 16. exp eye/ 17. 15 and 16 18. (trachoma$ or tracoma$ or trichiasis).tw. 19. exp entropion/ 20. entropion.tw. 21. or/18‐20 22. 13 or 14 or 17 or 21 23. exp ophthalmologic surgical procedures/ 24. exp eyelids/ 25. exp eyelid diseases/ 26. (surg$ or operat$ or incis$).tw. 27. (tars$ adj3 rotat$).tw. 28. (tars$ adj3 plate).tw. 29. (tarsotom$ or trabut or ballen or streatfield).tw. 30. (epilat$ or electrolysis or cryo$).tw. 31. ((tap$ or plaster$) adj5 eye$).tw. 32. ((tap$ or plaster$) adj5 lid$).tw. 33. or/23‐32 34. 22 and 33 35. 12 and 34 36. (201307$ or 201308$ or 201309$ or 201310$ or 201311$ or 201312$ or 2014$ or 2015$).ed. 37. 35 and 36

The search filter for trials at the beginning of the MEDLINE strategy is from the published paper by Glanville (Glanville 2006).

### Appendix 3. EMBASE (Ovid) search strategy

1. exp randomized controlled trial/ 2. exp randomization/ 3. exp double blind procedure/ 4. exp single blind procedure/ 5. random$.tw. 6. or/1‐5 7. (animal or animal experiment).sh. 8. human.sh. 9. 7 and 8 10. 7 not 9 11. 6 not 10 12. exp clinical trial/ 13. (clin$ adj3 trial$).tw. 14. ((singl$ or doubl$ or trebl$ or tripl$) adj3 (blind$ or mask$)).tw. 15. exp placebo/ 16. placebo$.tw. 17. random$.tw. 18. exp experimental design/ 19. exp crossover procedure/ 20. exp control group/ 21. exp latin square design/ 22. or/12‐21 23. 22 not 10 24. 23 not 11 25. exp comparative study/ 26. exp evaluation/ 27. exp prospective study/ 28. (control$ or prospectiv$ or volunteer$).tw. 29. or/25‐28 30. 29 not 10

### Appendix 4. ISRCTN search strategy

(Trachoma OR Tracoma OR Trichiasis)

### Appendix 5. ClinicalTrials.gov search strategy

(Trachoma OR Tracoma OR Trichiasis)

### Appendix 6. ICTRP search strategy

Trachoma OR Tracoma OR Trichiasis

## Data and analyses

**Comparison 1:** Bilamellar tarsal rotation versus tarsal advance and rotation

Outcome or subgroup title	No. of studies	No. of participants	Statistical method	Effect size
1 One or more lashes touching globe at nine months	2	260	Odds Ratio (M‐H, Fixed, 95% CI)	0.29 [0.16, 0.50]
2 Overcorrection following surgery	2	312	Odds Ratio (M‐H, Fixed, 95% CI)	2.57 [0.28, 23.25]
3 Defective lid closure following surgery	2	312	Odds Ratio (M‐H, Fixed, 95% CI)	1.90 [0.29, 12.37]

**Comparison 2:** Peri‐operative azithromycin versus no azithromycin

Outcome or subgroup title	No. of studies	No. of participants	Statistical method	Effect size
1 One or more lashes touching the globe at one year	3	1954	Odds Ratio (M‐H, Random, 95% CI)	0.85 [0.63, 1.14]
2 One or more lashes touching the globe at longer follow‐up	2	1589	Odds Ratio (M‐H, Random, 95% CI)	0.80 [0.60, 1.05]
2.1 Three years	1	1322	Odds Ratio (M‐H, Random, 95% CI)	0.76 [0.54, 1.07]
2.2 Four years	1	267	Odds Ratio (M‐H, Random, 95% CI)	0.87 [0.53, 1.41]

## Characteristics of studies

### Characteristics of included studies [ordered by study ID]

**Table:** Adamu 2002

Methods	Study Design: parallel group randomised trial of different operations for trichiasis. Eyes: for cases who only had trichiasis affecting one eye only that eye was included. In bilateral cases both eyes were included: surgery type was allocated randomly for the right eye and then the left eye was allocated to the other procedure. Analysis was not adjusted for bilateral cases.	
Participants	Country: Ethiopia Setting: teaching hospital Number of participants: 153 patients, 256 eyes Lost to follow up: 19 Male: 32, Female: 109 (gender unknown for 19 not followed up)	
Interventions	(1) Bilamellar tarsal rotation (2) Posterior lamellar tarsal rotation	
Outcomes	Primary outcome: recurrent trichiasis (one or more lashes touching eye) Secondary outcomes: overcorrection; complications Follow up: 3 months Reported improvement in vision, but no details given	
Notes	The unilamellar operation is different to the one described in Reacher 1990 and Reacher 1992 Funder: not specified	
***Risk of bias***		
**Bias**	**Authors' judgement**	**Support for judgement**
Random sequence generation (selection bias)	Unclear risk	An unspecified 'Lottery method' used. In bilateral cases the left eye always received the opposite treatment to that randomly allocated to the right eye.
Allocation concealment (selection bias)	Unclear risk	No description.
Blinding of participants and personnel (performance bias) All outcomes	Unclear risk	No description.
Blinding of outcome assessment (detection bias) All outcomes	Unclear risk	No description.
Incomplete outcome data (attrition bias) All outcomes	Unclear risk	No description of the individuals lost to follow‐up.
Selective reporting (reporting bias)	Unclear risk	No study protocol available. Limited information on analytical protocol in methods.

**Table:** Alemayehu 2004

Methods	Study Design: parallel group randomised trial of different levels of surgical training and background for surgeons performing trichiasis surgery Eyes: in bilateral cases both eyes were included. No adjustment was made in the analysis.	
Participants	Country: Ethiopia Setting: unclear Number of participants: 982 patients (1750 eyes) Lost to follow up: 464 eyes Female: 77.2% (absolute numbers not provided)	
Interventions	(1) Bilamellar tarsal rotation carried out by ophthalmologist (2) Bilamellar tarsal rotation carried out by integrated eye worker	
Outcomes	Primary outcome: recurrent trichiasis (one or more lashes touching eye) Secondary outcomes: severity of recurrence Follow up: 3 months	
Notes	Funder: not specified	
***Risk of bias***		
**Bias**	**Authors' judgement**	**Support for judgement**
Random sequence generation (selection bias)	Unclear risk	No description: "were randomised" but no further details provided.
Allocation concealment (selection bias)	Unclear risk	No description.
Blinding of participants and personnel (performance bias) All outcomes	Low risk	No masking of participants or surgical personnel, but unlikely to have influenced the outcome.
Blinding of outcome assessment (detection bias) All outcomes	Low risk	Follow‐up by ophthalmologists who were not involved in the surgery and masked to the allocation.
Incomplete outcome data (attrition bias) All outcomes	High risk	Participants were excluded post‐randomisation if surgery failed. 269 patients were lost to follow‐up at 3 months. .
Selective reporting (reporting bias)	Unclear risk	No study protocol available. Limited information on analytical protocol in methods.

**Table:** Bowman 2000

Methods	Study Design: cluster randomised study of surgery provided in the patients' village compared to surgery provided at a local health centre. Eyes: unclear how bilateral cases were analysed.	
Participants	Country: The Gambia Setting: village and health centre‐based surgery Number of participants: 8 pairs of villages (156 participants) Lost to follow up: 0 All participants had major trichiasis pre‐operatively	
Interventions	(1) Posterior lamellar tarsal rotation surgery in village (2) Posterior lamellar tarsal rotation surgery in health centre	
Outcomes	Primary Outcome: surgical uptake Secondary Outcomes: recurrent trichiasis, time taken from home to surgery, cost, complications Follow up: 1 week and 3 months	
Notes	Funder: Sight Savers International	
***Risk of bias***		
**Bias**	**Authors' judgement**	**Support for judgement**
Random sequence generation (selection bias)	Low risk	Quote: "Clusters were paired by district or geographical proximity. One cluster from each pair was randomly allocated to receive village‐based surgery and the other, health centre‐based surgery." Method of sequence generation not specified.
Allocation concealment (selection bias)	Low risk	All clusters randomly allocated at baseline, before the TT patients were identified.
Blinding of participants and personnel (performance bias) All outcomes	Low risk	Participants and personnel not masked to community allocation. Unlikely to have influenced the outcome.
Blinding of outcome assessment (detection bias) All outcomes	Unclear risk	No description.
Incomplete outcome data (attrition bias) All outcomes	Low risk	Data complete.
Selective reporting (reporting bias)	Low risk	No study protocol available. Clear analytical approach in methods.

**Table:** Burton 2005a

Methods	Study Design: parallel group randomised trial of peri‐operative azithromycin treatment Eyes: only one eye per participant was included in the analysis. In bilateral cases, the eye with more severe baseline trichiasis was included.	
Participants	Country: The Gambia Setting: health centres Number of participants: 451 Lost to follow up: 25 Male: 133, Female: 318 A consecutive, geographically defined sub‐set of the original participants was followed‐up at 4 years. Number of participants: 356 Lost to follow up: 90 Male: 103, Female: 253 Major trichiasis cases only	
Interventions	(1) Posterior lamellar tarsal rotation surgery (no placebo) (2) Posterior lamellar tarsal rotation surgery followed by single‐dose azithromycin treatment at baseline and 6 months, administered to the patient and the members of their household	
Outcomes	Primary Outcome: recurrent trichiasis by one year Secondary Outcomes: visual acuity by reduced LogMAR chart, conjunctival infection, corneal opacification Follow up: 12 months and 4 years (subset)	
Notes	Funder: International Trachoma Initiative	
***Risk of bias***		
**Bias**	**Authors' judgement**	**Support for judgement**
Random sequence generation (selection bias)	Low risk	Quote "Separate randomisation sequences were generated for each surgeon from random number tables and blocked in groups of four"
Allocation concealment (selection bias)	Low risk	Quote "Treatment allocations were concealed in opaque sequentially numbered envelopes"
Blinding of participants and personnel (performance bias) All outcomes	Low risk	Quote "Immediately following surgery a nurse not involved in clinical assessments or surgery administered the randomly allocated treatment." Participants were not masked to allocation, as no placebo was used.
Blinding of outcome assessment (detection bias) All outcomes	Low risk	Quote "Examiners were ‘‘blinded’’ to treatment allocation."
Incomplete outcome data (attrition bias) All outcomes	Low risk	98% follow‐up of those still alive at 1 year. At 4 years 94% of the surviving patients were re‐examined
Selective reporting (reporting bias)	Low risk	Clear analytical approach in methods.

**Table:** Dhaliwal 2005

Methods	Study Design: parallel groups randomised to one of three procedures Eyes: in bilateral cases both eyes were separately randomised. No adjustment was made for this in the analysis.	
Participants	Country: India Setting: hospital ophthalmology department Number of participants: 77 patients (90 eyes) Lost to follow up: 0 eyes Female: unclear as % is provided by eye and not person	
Interventions	(1) Terminal tarsotomy and rotation (2) Tarsal advance and rotation (3) Tarsal grooving	
Outcomes	Post‐opearative trichiasis and/or entropion at 6 months	
Notes	Terminal tarsotomy and rotation is different from the PLTR/Trabut Funder: not specified	
***Risk of bias***		
**Bias**	**Authors' judgement**	**Support for judgement**
Random sequence generation (selection bias)	Unclear risk	Method for the allocation for the second eye is unclear and excluded the method used on the first eye to be operated.
Allocation concealment (selection bias)	Unclear risk	No description.
Blinding of participants and personnel (performance bias) All outcomes	Unclear risk	No description.
Blinding of outcome assessment (detection bias) All outcomes	Unclear risk	No description.
Incomplete outcome data (attrition bias) All outcomes	Low risk	High follow‐up.
Selective reporting (reporting bias)	Low risk	No study protocol available. Clear analytical approach in methods.

**Table:** Gower 2013

Methods	Study Design: parallel group randomised controlled trial bilamellar tarsal rotation surgery with or without a TT clamp Eyes: both eyes of bilateral cases were included in the analysis. Appropriate adjustment was made in the analysis for bilateral cases.	
Participants	Country: Tanzania Setting: health centres Number of participants: 1917 Number of Eyes: 3345 Lost to follow up: 51 Male: 490, Female: 1427	
Interventions	(1) Standard bilamellar tarsal rotation (2) Bilamellar tarsal rotation performed with a TT clamp	
Outcomes	Primary Outcome: was defined as one or more of the following: recurrent trichiasis, eyelid contour abnormality or granuloma. Secondary outcomes: post‐operative trichiasis, eyelid contour abnormality or granuloma individually. Follow‐up: 6 weeks, 12 months, 24 months.	
Notes	18 different surgical technicians performed the surgery. Surgeons were randomly allocated at the outset to only perform one of the two types of operation. Each recruitment day there were 4 surgical technicians operating – 2 standard procedure and 2 with the TT clamp. Patients with TT were randomly allocated to one of the 4 surgical technicians operating. Funder: Bill and Melinda Gates Foundation	
***Risk of bias***		
**Bias**	**Authors' judgement**	**Support for judgement**
Random sequence generation (selection bias)	Low risk	Quote "Randomization assignments were created using permuted block sizes of 6 to 12".
Allocation concealment (selection bias)	Low risk	Quote "Randomization assignments were placed in opaque envelopes and stored in a locked office until distribution".
Blinding of participants and personnel (performance bias) All outcomes	Low risk	Participants and surgical technicians were not masked to the allocation; however, this is unlikely to have affected the outcome measure.
Blinding of outcome assessment (detection bias) All outcomes	Low risk	Quote "Examinations were conducted by a single trained examiner who was not involved in enrolment, randomization, or surgical procedures".
Incomplete outcome data (attrition bias) All outcomes	Low risk	98% follow‐up rate.
Selective reporting (reporting bias)	Low risk	No study protocol available. Clear analytical approach in methods.

**Table:** Graz 1999

Methods	Study Design: parallel group randomised trial of epilation versus sticking tape. Eyes: in bilateral cases both eyes were treated the same way and the data from both included in the analysis. The authors combined the data from both eyes to give a single 'clinical status' outcome.	
Participants	Country: China Setting: hospital and home Number of participants: 57 (68 eyes) Lost to follow up: 0 Male: 20, Female: 37	
Interventions	(1) Epilation alone for 12 weeks (2) Sticking plaster for 12 weeks (3) Sticking plaster for 8 weeks, followed by cross‐over to epilation	
Outcomes	Successful outcome: no lashes touching the eye. Secondary outcomes: visual acuity by Snellen E‐chart, corneal status, patient‐reported symptoms by questionnaire. Follow up: 3 months	
Notes	The group that had sticking plaster for 8 weeks followed by epilation has been excluded from our analysis as this group received both interventions. Funder: Swiss Agency for Development and Cooperation	
***Risk of bias***		
**Bias**	**Authors' judgement**	**Support for judgement**
Random sequence generation (selection bias)	Unclear risk	No description.
Allocation concealment (selection bias)	Unclear risk	No description.
Blinding of participants and personnel (performance bias) All outcomes	Unclear risk	Personnel not masked; performance risk possible.
Blinding of outcome assessment (detection bias) All outcomes	High risk	Observer not masked. Quote "A blinded design was not possible for obvious reasons, but data entry and analysis were blinded."
Incomplete outcome data (attrition bias) All outcomes	Low risk	Data complete.
Selective reporting (reporting bias)	Low risk	Data complete.

**Table:** Rajak 2011a

Methods	Study Design: parallel group randomised trial of epilation versus surgery for minor trichiasis. Non‐inferiority trial. Eyes: only one eye per participant was included in the analysis. In bilateral cases, one eye was randomly assigned to be the 'study eye', although both eyes were treated, if indicated.	
Participants	Country: Ethiopia Setting: health centres Number of participants: 1300 Lost to follow up: 81 Male: 537, Female: 863 Minor trichiasis cases only	
Interventions	(1) Posterior lamellar tarsal rotation (2) Epilation with new forceps and training	
Outcomes	Primary Outcome: proportion of individuals at any follow‐up who had “failed” defined as either (1) five or more eyelashes touching the globe or (2) a history of surgery performed in the trial eye at any point during the follow‐up period (in the case of the surgical arm this would be repeat surgery). A pre‐specified non‐inferiority margin was 10%. Secondary Outcomes: corneal opacity change, visual acuity by LogMAR E‐chart, entropion, conjunctival inflammation and symptoms by questionnaire. Follow up at 6, 12, 18 and 24 months.	
Notes	Funder: Band Aid Foundation and The Wellcome Trust. Johnson & Johnson donated all sutures. Tweezerman donated all forceps.	
***Risk of bias***		
**Bias**	**Authors' judgement**	**Support for judgement**
Random sequence generation (selection bias)	Low risk	Quote "Participants were randomly allocated to the epilation or surgery groups using a 1:1 allocation ratio for each surgeon, using a computer‐generated randomisation sequence with random block sizes. Randomisation was stratified by surgeon because of possible intersurgeon variability."
Allocation concealment (selection bias)	Low risk	Quote "The random allocation sequences for each surgeon were concealed in sequentially numbered, sealed, opaque envelopes, which were colour coded for surgeon and placed in separate containers for each surgeon. The person who prepared these envelopes was independent of all other aspects of the trial."
Blinding of participants and personnel (performance bias) All outcomes	Low risk	Participants were not masked to their allocation, as they either received surgery or practiced epilation. Other than the surgeons performing the operation and the epilation trainer, all other personnel were masked to the allocation.
Blinding of outcome assessment (detection bias) All outcomes	Low risk	Quote "The two individuals responsible for all the clinical outcome measurements were masked to the allocation. At follow‐up, the trichiasis and corneal examination was performed and recorded before the eyelid was everted, so that the examiner was masked to whether surgery had been performed on the tarsal conjunctiva."
Incomplete outcome data (attrition bias) All outcomes	Low risk	93.5% follow‐up at 24 months.
Selective reporting (reporting bias)	Low risk	Study protocol published online.

**Table:** Rajak 2011b

Methods	Study Design: parallel group randomised trial of absorbable versus silk sutures for major trichiasis. Eyes: only one eye per participant was included in the analysis. In bilateral cases, one eye was randomly assigned to be the 'study eye', although both eyes were treated, if indicated.	
Participants	Country: Ethiopia Setting: health centres Number of participants: 1300 Lost to follow up: 82 Male: 285, Female: 1015	
Interventions	(1) Posterior lamellar tarsal rotation with absorbable sutures (2) Posterior lamellar tarsal rotation with silk sutures	
Outcomes	Primary Outcome: proportion of those individuals seen at the 12‐month follow‐up who were found to have either (1) recurrent trichiasis, defined as one or more lashes touching the eye or clinical evidence of epilation; or (2) a history of repeat TT surgery during the first year. Secondary Outcomes: corneal opacity change, visual acuity by LogMAR E‐chart, entropion, conjunctival inflammation and symptoms by questionnaire. Follow up at 6, 12, 18 and 24 months.	
Notes	Funder: Band Aid Foundation and The Wellcome Trust. Johnson & Johnson donated all sutures.	
***Risk of bias***		
**Bias**	**Authors' judgement**	**Support for judgement**
Random sequence generation (selection bias)	Low risk	Quote "Participants were randomly allocated to the silk or polyglactin‐910 suture arms using a 1:1 allocation ratio for each surgeon, using a computer‐generated randomisation sequence with random block sizes. Randomisation was stratified by surgeon because of possible inter‐surgeon variability."
Allocation concealment (selection bias)	Low risk	Quote "The random allocation sequences for each surgeon were concealed in sequentially numbered, sealed, opaque envelopes, which were placed in separate containers for each surgeon; a person independent of all other aspects of the trial prepared these envelopes."
Blinding of participants and personnel (performance bias) All outcomes	Low risk	Quote "Participants and surgeons were aware of the suture allocation." Other personnel involved in the study were masked to the allocation.
Blinding of outcome assessment (detection bias) All outcomes	Low risk	Quote "The two individuals who were responsible for all the clinical observations in this trial were masked to the allocation."
Incomplete outcome data (attrition bias) All outcomes	Low risk	95.1% follow‐up at 12 months.
Selective reporting (reporting bias)	Low risk	Study protocol published online.

**Table:** Reacher 1990

Methods	Study Design: parallel group randomised trial of five different operations for trachomatous trichiasis. Eyes: only one eye per participant was included in the analysis. Unclear how lids were selected in bilateral cases.	
Participants	Country: Oman Setting: not specified Number of participants: 165 (165 eyes) Lost to follow‐up: 12 Gender: not specified	
Interventions	(1) Bilamellar tarsal rotation (2) Tarsal advance and rotation (3) Eversion splinting (4) Tarsal advance (5) Tarsal grooving	
Outcomes	Primary outcome: "treatment success" defined as no eyelash/eyeball contact in the primary position and complete eyelid closure. Mean follow up: 7.9 months. A follow‐up schedule is not specified beyond 14 days.	
Notes	Three surgeons performed the surgery, with two surgeons performing relatively few of the operations. Funder: not specified	
***Risk of bias***		
**Bias**	**Authors' judgement**	**Support for judgement**
Random sequence generation (selection bias)	Unclear risk	Limited information. Quote "operations allocated by random number tables".
Allocation concealment (selection bias)	Unclear risk	No comment on concealment of the random sequence.
Blinding of participants and personnel (performance bias) All outcomes	Unclear risk	Participants and surgeon were not masked to the allocation.
Blinding of outcome assessment (detection bias) All outcomes	Unclear risk	Not specified.
Incomplete outcome data (attrition bias) All outcomes	Low risk	93% follow‐up rate.
Selective reporting (reporting bias)	Low risk	No study protocol available. Clear analytical approach in methods.

**Table:** Reacher 1992

Methods	Study Design: three separate parallel group trials for minor trichiasis, major trichiasis and defective lid closure.	
Participants	Country: Oman Setting: hospital or health centre Number of participants: 367 (172 eyes with minor trichiasis, 200 eyes with major trichiasis, 21 eyes with lid closure defect) Lost to follow up: 24	
Interventions	Minor trichiasis: (1) Bilamellar tarsal rotation (2) Electrolysis (3) Cryotherapy Major trichiasis: (1) Bilamellar tarsal rotation (2) Tarsal advance and rotation Defective lid closure: (1) Tarsal advance and rotation (2) Tarsal advance with graft	
Outcomes	Primary outcome: "Successful outcome" defined as no eyelash/eyeball contact in the primary position, complete eyelid closure, no overcorrection and a cosmetically acceptable outcome.	
Notes	The group with lid closure defects was too small to be analysed separately and has been excluded from the analysis. Funder: Edna McConnell Clarke Foundation and National Institutes of Health.	
***Risk of bias***		
**Bias**	**Authors' judgement**	**Support for judgement**
Random sequence generation (selection bias)	Low risk	Quote: "Allocation was made by opening a sequence of sealed envelopes specific for lid grade that contained one of a random series of operations generated by computer."
Allocation concealment (selection bias)	Low risk	Quote: "Allocation was made by opening a sequence of sealed envelopes specific for lid grade that contained one of a random series of operations generated by computer."
Blinding of participants and personnel (performance bias) All outcomes	Unclear risk	Participants and surgeons were not masked to the allocation.
Blinding of outcome assessment (detection bias) All outcomes	Low risk	Masked independent assessor.
Incomplete outcome data (attrition bias) All outcomes	Unclear risk	96% follow‐up, but very variable time frames (1.2 to 25 months).
Selective reporting (reporting bias)	Low risk	No study protocol available. Clear analytical approach in methods.

**Table:** West 2006

Methods	Design: parallel group randomised trial of peri‐operative azithromycin treatment. Eyes: only one eye per participant was included in the analysis. In bilateral cases, the study eye was determined by the study number (left eye if an odd number).	
Participants	Country: Ethiopia Setting: health centres Number of participants: 1452 Lost to follow‐up: participants were followed until they developed recurrence or until 1 year post‐operatively, whichever came first. 98% of eligible subjects were followed at 1 year. Male: 331, Female: 1121 Number of participants: 1452 Lost to follow‐up: 30 Male: 331, Female: 1121	
Interventions	(1) Bilamellar tarsal rotation with tetracycline eye ointment (2) Bilamellar tarsal rotation with azithromycin to the patient only (3) Bilamellar tarsal rotation with azithromycin to the patient and their family	
Outcomes	Primary Outcome: post‐operative trichiasis 1 year and 3 years after surgery Secondary Outcomes: visual acuity reported in a subset of the trial participants	
Notes	Funder: National Eye Institute.	
***Risk of bias***		
**Bias**	**Authors' judgement**	**Support for judgement**
Random sequence generation (selection bias)	Low risk	Random sequence, with variable block size.
Allocation concealment (selection bias)	Low risk	Allocation concealed in envelopes.
Blinding of participants and personnel (performance bias) All outcomes	Low risk	Participants were not blinded to the allocation.
Blinding of outcome assessment (detection bias) All outcomes	Low risk	Outcome assessments were performed by a masked grader.
Incomplete outcome data (attrition bias) All outcomes	Low risk	98% follow‐up at one year; 91.1% follow‐up at three years.
Selective reporting (reporting bias)	Low risk	Study protocol published and no evidence of selective reporting of outcomes

**Table:** Zhang 2006

Methods	Design: parallel group randomised trial of peri‐operative azithromycin treatment. Eyes: bilateral cases both eyes included.	
Participants	Country: Nepal Setting: field camps Number of participants: 109 (148 eyes operated) Lost to follow‐up: uncertain, as reported by eye rather than person Male: 29, Female: 80	
Interventions	(1) Bilamellar tarsal rotation with placebo (2) Bilamellar tarsal rotation with azithromycin, single dose	
Outcomes	Primary outcome: recurrent trichiasis (1+ lash touching the eye)	
Notes	Funder: Fight for Sight, International Trachoma Initiative, National Institutes of Health.	
***Risk of bias***		
**Bias**	**Authors' judgement**	**Support for judgement**
Random sequence generation (selection bias)	High risk	No random sequence. Patients allocated to azithromycin and placebo on an alternating basis.
Allocation concealment (selection bias)	High risk	No concealment.
Blinding of participants and personnel (performance bias) All outcomes	Low risk	Placebo used.
Blinding of outcome assessment (detection bias) All outcomes	Low risk	Examinations by masked graders.
Incomplete outcome data (attrition bias) All outcomes	Unclear risk	Some minor internal inconsistencies data.
Selective reporting (reporting bias)	High risk	Excluded "surgical failures" from the analysis, defined as five+ lashes touching the globe at 3 months.

TT = trachomatous trichiasis

## Differences between protocol and review

The protocol for this review was originally published in 2003 (Yorston 2003). Since that time Cochrane methods have changed and we have incorporated these changes, in particular assessment of risk of bias in included studies.
